# Alveolar epithelial cells are competent producers of interstitial extracellular matrix with disease relevant plasticity in a human in vitro 3D model

**DOI:** 10.1038/s41598-023-35011-z

**Published:** 2023-05-31

**Authors:** Oskar Rosmark, Måns Kadefors, Göran Dellgren, Christofer Karlsson, Anders Ericsson, Sandra Lindstedt, Johan Malmström, Oskar Hallgren, Anna-Karin Larsson-Callerfelt, Gunilla Westergren-Thorsson

**Affiliations:** 1 Lung Biology, Department of Experimental Medical Science, Lund University, BMC C12, 22184 Lund, Sweden; 2 Transplant Institute and Department of Cardiothoracic Surgery, Sahlgrenska University Hospital, Gothenburg, Sweden; 3 Division of Infection Medicine, Department of Clinical Sciences, Lund University, Lund, Sweden; 4 Blekinge Hospital, Karlskrona, Sweden; 5 Department of Thoracic Surgery, Lund University, Lund, Sweden; 6 Division of Respiratory Medicine and Allergology, Department of Clinical Sciences, Lund University, Lund, Sweden

**Keywords:** Respiratory tract diseases, Chronic obstructive pulmonary disease, Experimental models of disease, Respiration, Respiratory system models, Biological techniques, Cell biology, Molecular biology, Diseases, Molecular medicine, Pathogenesis

## Abstract

Alveolar epithelial cells (AEC) have been implicated in pathological remodelling. We examined the capacity of AEC to produce extracellular matrix (ECM) and thereby directly contribute towards remodelling in chronic lung diseases. Cryopreserved type 2 AEC (AEC2) from healthy lungs and chronic obstructive pulmonary disease (COPD) afflicted lungs were cultured in decellularized healthy human lung slices for 13 days. Healthy-derived AEC2 were treated with transforming growth factor ß1 (TGF-β1) to evaluate the plasticity of their ECM production. Evaluation of phenotypic markers and expression of matrisome genes and proteins were evaluated by RNA-sequencing, mass spectrometry and immunohistochemistry. The AEC2 displayed an AEC marker profile similar to freshly isolated AEC2 throughout the 13-day culture period. COPD-derived AECs proliferated as healthy AECs with few differences in gene and protein expression while retaining increased expression of disease marker HLA-A. The AEC2 expressed basement membrane components and a complex set of interstitial ECM proteins. TGF-β1 stimuli induced a significant change in interstitial ECM production from AEC2 without loss of specific AEC marker expression. This study reveals a previously unexplored potential of AEC to directly contribute to ECM turnover by producing interstitial ECM proteins, motivating a re-evaluation of the role of AEC2 in pathological lung remodelling.

## Introduction

The structure of the alveolar lung compartment maximizes the interface for gas exchange. Disruption of this architecture is a fundamental part of both chronic obstructive lung disease (COPD), in the form of emphysema, and of lung fibrosis where idiopathic pulmonary fibrosis (IPF) is the most common form. Remodelling of lung tissue is a complex process where the proportions as well as the structural organization of ECM are altered over the course of years in chronic lung diseases^1,2^. The pathophysiological processes behind this remodelling are unfortunately not fully understood, which hampers the development of new efficient treatment options.

The alveolar type II cells (AEC2) are known as important producers of surfactants to maintain alveolar structures during ventilation and also function as self-renewing progenitor cells to alveolar type I cells^3^. Studies of pathological ECM remodelling in the lung have previously focused on mesenchymal cells as the effector cells responding to signals from epithelial cells^4^. AEC2 has been proposed to be central drivers in the development of lung fibrosis, with dysfunctional AEC2 promoting activation of fibroblasts and subsequent tissue remodelling^5^. AEC2 senescence is proposed to be part of this pathology, which has also been observed in emphysematous lungs^6^. Transforming growth factor ß-1 (TGF-β1) is recognized as a crucial inducer of ECM deposition and fibrotic lung remodelling. AEC2 cells have been shown to be important producers of TFG-ß1 in IPF, which foster a profibrotic response in fibroblasts^7^. As dysfunction of AEC2 appears to be central to two superficially opposite pathological processes leading to remodelling of alveolar architecture, AEC may actually play a more direct role in these processes by altering the composition of the ECM. We aimed to examine if the ECM production of AEC2 might stretch beyond maintaining their own basement membrane (BM).

In vitro studies of alveolar epithelial cells, both type I cells (AEC1) and AEC2, pose considerable challenges, as AEC1 do not readily expand in in vitro culture and AEC2 tends to rapidly lose its phenotype in 2D cultures on plastic surfaces^8^. Furthermore, parenchymal lung cells are not readily available from human subjects, and up until recently, there were no available specific surface markers for these cells. We utilized the AEC2 specific marker HT2-280^9^ to isolate human AEC2 from lungs without lung disease and from COPD patients and frozen them without prior expansion on tissue culture plastic. The cryopreserved AEC2 were then seeded directly into decellularized non-diseased human lung tissue slices to maintain as much as possible of their native cell phenotype. Finally, we added a pro-fibrotic stimulus in the form of TGF-β1 to study the plasticity in the ECM expression capacity of these cells.

Gene expression of the matrisome was evaluated with RNA sequencing and the protein production of the AEC was analysed with mass spectrometry using a labelling of cell derived proteins with amino acids containing stable heavy isotopes, to separate the newly produced proteins from the ECM background provided by the human lung slice^10^. Our results show that AEC expresses not only a wide range of BM proteins, but also a considerable number of what can be considered interstitial ECM components, traditionally associated to mesenchymal cells. AEC2 from lungs of end stage COPD patients resembled healthy AEC2 when cultured in a healthy derived ECM substrate, while TGF-β1 stimuli induced a pronounced increase in interstitial matrix production from healthy AEC2. Our data reveal AEC2 as competent ECM producers with the capacity to play a direct role in remodelling processes in chronic lung disease, adding a new perspective in the understanding of alveolar ECM homeostasis and the pathogenesis of destructive alveolar remodelling.

## Materials and methods

### Patient material

Human lung material was collected from COPD explant lungs and lungs from organ donors without any known lung disease which were not suitable for transplantation. Tissue was acquired from Sahlgrenska University hospital in Gothenburg and Skåne University Hospital in Lund. In addition, cells were isolated from tumour free resection material from one patient with COPD treated for lung cancer at Blekinge hospital, Karlskrona. Informed written consent was obtained from all patients. The use of human material in this study was approved by the Swedish ethical committees in Lund and Gothenburg (Dnr. 2006/91, 2008/413, 2015/891 and 2016/2, 2018/869), all experimental procedures were performed in accordance with the approved guidelines of the ethical committee. Patient characteristics and allocation of material for decellularized lung slices (DLS) and/or cell preparation are presented in Supplementary Table 1.

### Generation of decellularized human lung slices

Decellularized human lung slices (DLS) were produced as previously described^10^. In short, 1 cm^3^ cubes of pleura adjacent lung tissue was snap frozen and stored at -80 °C before cryosectioning to a thickness of 350 µm. Slices were decellularized by a 4-h exposure to serial changes of a detergent solution containing 8 mM 3-((3-cholamidopropyl) dimethylammonio)-1-propanesulfonate (CHAPS), 1 M NaCl, 25 mM EDTA in phosphate buffered saline (PBS) at pH 8.0. Lastly slices were treated with 90 U/mL of turbonuclease (Sigma-Aldrich, Saint Louis, Missouri, USA), before repeated rinses with PBS and storage at 4 °C in PBS with 100 units/mL penicillin, 100 µg/mL streptomycin and 250 ng/mL Amphotericin B (Sigma-Aldrich, Saint Louis, MO, USA) for up to 3 weeks before use in cell culture.

### Cell isolation

AEC2 were isolated from parenchymal lung tissue using the cell surface marker HT2-280. Lung parenchyma was dissected into pieces of ~ 30 mm^3^, removing macroscopically visible airways, blood vessels and pleura. The tissue pieces were rinsed three times with cold PBS, cut with scissors into ~ 2 mm^3^ pieces and digested for 1.5 h in enzyme buffer containing 300 units/mL Collagenase Type 1 (Gibco, Dublin, Ireland), 1000 units/mL of Hyaluronidase (Serva, Heidelberg, Germany) and 100 Kunitz units/mL of Deoxyribonuclease I (Sigma-Aldrich). The digest was passed through a 100 µm cell strainer (Pluriselect, Leipzig, Germany), treated with red cell lysis buffer, washed in PBS, incubated with anti-HT2-280 antibody (Terrace Biotech, San Francisco, CA, USA) followed by anti-mouse IgM microbeads (Miltenyi Biotec, Bergisch Gladbach, Germany). Positive selection of labelled cells was performed with LS Columns (Miltenyi Biotec). Freshly isolated cells were frozen at a concentration of 0,5–1*10^6^ cells/mL in 7.5% v/v DMSO (Sigma-Aldrich), 30% Fetal Bovine Serum (Gibco), 17.5% DPBS (Sigma-Aldrich), 0.1% Heparin (Leo Pharma, Ballerup, Denmark) and 45% DMEM (Gibco). Cells were stored at − 150 °C for up to 2 years before use in cell culture.

### Flow cytometry

Cryopreserved HT2-280 positive cells from four healthy control cell donors and two of the COPD donors were thawed, potential Fc-receptors were blocked with Human Fc Block (BD Pharmingen, San Diego, CA, USA). Staining was performed using BD Cytofix/Cytoperm Fixation/Permeabilization Solution Kit (BD Pharmingen), according to the manufacturer’s instructions. Cells were stained with anti-pro-surfactant protein C (pro-SPC) antibody (ab90716, Abcam, Cambridge, UK) followed by an Alexa Fluor Plus 647 conjugated secondary antibody. Cells stained with primary and secondary antibodies and control cells stained only with secondary antibody were acquired on a BD LSR II cytometer (BD Pharmingen) and analyzed using FlowJo software version 10.8.0 (BD Bioscience).

### Cell culture with stable isotope labelled amino acids

Cell culture was performed as four individual experiments over a period of 10 weeks. In each experiment, cells from two donors (healthy or COPD) were cultured in DLS from four different donors for healthy cells and three different donors for COPD cells (Supplementary Table 2). The three COPD cell donors were spread across three separate experiments. DLS were pre-incubated with complete cell culture medium at 37 °C and 5% CO_2_ for 1–2 h before cell seeding. Culture medium consisted of Small Airway Epithelial Cell Growth Medium (Promocell, Heidelberg, Germany) modified to be Lysine and Arginine free, supplemented with 1% dialyzed Fetal bovine serum (GIBCO, ref: A3382001), 0,5 mg/mL 4,4,5,5-D4 L-lysine, 0,5 mg/mL 13C6 L-arginine (Thermo Fisher Scientific, Waltham, MA, USA), 100 units/mL penicillin, 100 µg/mL streptomycin and 250 ng/mL Amphotericin B (Sigma-Aldrich). The stable heavy isotope labelled L-Lysine and L-arginine were added to distinguish between newly synthesized proteins by the AEC (Cell derived) and pre-existing proteins originating from the DLS in the mass spectrometry analysis. Cell suspensions containing 181,000–250,000 cells, the number depending on cell availability, were added to each well of 12-well suspension culture plates (Sarstedt, Nümbrecht, Germany) with DLS up to a total medium volume of 1 mL/well. Medium without any cells was added to wells with control DLS. Plates with seeded slices were incubated on an orbital shaker (19 mm orbit, 100 rpm) for the first 24 h to increase cell attachment to DLS, and slices were then transferred to wells of new plates with fresh culture medium to minimize any effect of unattached cells in subsequent analyses. Culture medium was then replaced every 72 h until termination of the experiment, 13 days after initial cell seeding. At the medium changes day seven and ten, 2 ng/mL of recombinant human TGF-β1 (R&D systems, Minneapolis, MN, USA) was added to a subset of wells containing cells from healthy donors. Samples for RNA sequencing and MS was collected at day seven and 13 of culture by rinsing lung slices in DPBS before dividing them in two with a scalpel, placing one of the pieces in 100 µL of RNAlater (Sigma-Aldrich), before storage of both pieces at − 80 °C.

### Resazurin assay for metabolic activity

At each change of culture medium from day 4 of culture and onwards, the spent culture medium was replaced with 500 µL of complete culture medium containing 10% HS presto blue cell viability reagent (Thermo Fisher Scientific). Slices were incubated for 1 h before collecting the assay medium and replacing it with 1 mL/well of fresh culture medium and returning plates to the incubator. The assay medium was analysed by measuring the absorbance at 570 and 600 nm, using complete culture medium with 10% assay reagent as a reference. The percentage of resazurin reduction was calculated according to manufacturer’s instructions. Statistical testing for differences between groups was performed using a linear mixed model using the lme4^11^ for package in R (version 4.1.0), with DLS and cell donors specified as random effects, a p-value < 0,05 was considered statistically significant. Visualization of model results was performed using the sjPlot R package^12^.

### RNA sequencing

Samples for RNA sequencing were shipped frozen in RNAlater to Genewiz (South Plainfield, NJ, USA) for RNA extraction and sequencing. RNA extraction was performed using RNeasy Plus Mini Kit (Qiagen, Venlo, Netherlands). RNA quantity and integrity were evaluated with Nanodrop 2000 (Thermo Fisher Scientific), Qubit RNA (Thermo Fisher Scientific) and TapeStation (Agilent technologies, Santa Clara, CA, USA). One sample (healthy day 7) had both low RNA quantity and a low DV200 (26%) and was therefore excluded. The remaining 71 samples had an average DV200 value of 78% (range 55–90%). cDNA synthesis and mRNA library preparation were performed using Illumina Stranded mRNA Prep (Illumina, San Diego, CA, USA). Sequencing was performed on an Illumina HiSeq (2 × 150 bp). The data generated in this study have been deposited in NCBI's Gene Expression Omnibus^13^ and are accessible through GEO Series accession number GSE191279.

### Bioinformatics analysis of RNA-sequencing data

Sequence reads were trimmed using Trimmomatic v.0.36. The trimmed reads were mapped to the Homo sapiens GRCh38 reference genome available on ENSEMBL using the STAR aligner v.2.5.2b. Unique gene hit counts were calculated by using featureCounts from the Subread package v.1.5.2. The hit counts were summarized and reported using the gene_id feature in the annotation file. Only unique reads within exon regions were counted. Data containing raw read counts were analysed using the DESeq2^14^ package in R (version 4.1.0). One sample (healthy day 7) with low read counts was excluded from the analysis. Genes with a minimum of 1 CPM in at least 75% of the samples were used for testing for differentially expression genes. The cell group (COPD, Healthy or TGF-β1) in combination with the culture day (7 or 13) was used to specify five levels (e.g. COPD day 7) which were used as an explanatory variable, with the DLS donor and scaled and centred resazurin reduction values as additional explanatory variables. The full model was used for all differential expression analysis, specifying contrast for the comparison of interest. The Benjamini–Hochberg adjusted p-value cutoff was set to 0.05.

For evaluation of differentiation markers, five publicly available datasets were reanalyzed. To reduce the potential impact of different bioinformatical processing when comparing these datasets to our data, raw sequencing data (FASTQ files) were re-analyzed following the same workflow used for the original data generated in this study. Adaptor sequences were trimmed using^15^ v0.38 and aligned to GRCh Build 38^16^ v2.5.2b generating gene count data used for subsequent analysis. One of the reference datasets, GSE173738 contains data from mesenchymal cells isolated from some of the same healthy lung donors used in this study, of which CD90^+^CD13^+^ cell samples were^17^. Two datasets contain data from freshly isolated HT2-280 positive AEC2 cells, GSE166703 have cells from three male patients with no history of lung disease or smoking, age 47, 52 and 61 (Dr A. Waghray, personal communication)^18^ from three individuals 43, 62 and 77 years old. Two datasets contain data from AEC2 derived organoid cultures, generated from HT2-280^+^ sorted AEC2 after 12–14 days organoid culture^19^, and EpCAM^+^ sorted cells from distal lung tissue after 10–15 days (organoid establishment with stromal support cells) plus an additional 1–3 days of organoid culture^20^. As a reference for matrisome^21^ was used, extracting gene expression counts from normal lungs from 18 subjects > 20 years of age. The material came from homogenized lung tissue without any cell selection. Heatmaps were created with the pheatmap^22^, clustering was performed using the complete linkage clustering option.

### Sample preparation for mass spectrometry

Frozen samples were transferred to 250 µL of 8 M urea, 100 mM ammonium bicarbonate in liquid chromatography grade H_2_O and homogenized using an Omni Tissue Homogenizer equipped with hard tissue tips (Omni International, Kennesaw, GA, USA). The homogenate was centrifuged at 14000 × *g* for 15 min and the protein concentration of the supernatant was estimated using a Nanodrop 2000. 20 µg of protein from each sample was further processed by in-solution digestion. Samples were reduced in 5 mM TCEP for 60 min 37 °C, alkylated in 10 mM Iodoacetic acid for 30 min at room temperature, and digested with 10 ng/µL of Trypsin/Lys-C (Thermo Fisher Scientific) at 37 °C for 14 h. The digestion was stopped by acidification with formic acid (FA) to a pH of ~ 3. Samples were desalted using C18 columns (Macro spin column, Harward apparatus, Holliston, MA, USA), vacuum concentrated to until dry and resuspended in 2% acetonitrile with 0.2% FA in liquid chromatography grade H2O. Peptide concentration was determined using a colorimetric peptide Assay (Thermo Fisher Scientific).

### Liquid chromatography mass spectrometry

Analyses were performed on a Q Exactive HF-X mass spectrometer (Thermo Fisher Scientific) connected to an EASY-nLC 1200 ultra-high-performance liquid chromatography system (Thermo Fisher Scientific). Peptides were trapped on pre-column (PepMap100 C18 3 µm; 75 µm × 2 cm, Thermo Fisher Scientific) and separated on an EASY-Spray column (ES903, column temperature 45 °C, Thermo Fisher Scientific). Equilibrations of columns and sample loading were performed per manufacturer’s guidelines. Solvent A was used as stationary phase (0.1% formic acid), and solvent B (mobile phase; 0.1% formic acid, 80% acetonitrile) was used to run a linear gradient from 5 to 38% over 120 min at a flow rate of 350 nL/min.

Data dependent acquisition was performed with one full MS scan (resolution 60,000 @ 200 m/z; mass range 350–1650 m/z) followed by MS/MS scans (resolution 15,000 @ 200 m/z) of the 20 most abundant ion signals. The precursor ions were isolated with a 1.6 m/z width and fragmented using higher-energy collisional-induced dissociation at a normalized collision energy of 27%. Charge state screening was enabled and unassigned or singly charged ions were rejected. The dynamic exclusion window was set to 15 s. Only MS precursors that exceeded a threshold of 8e^3^ were allowed to trigger MS/MS scans. The ion accumulation time (IT) was set to 100 ms (MS) and 30 ms (MS/MS) using an automatic gain control (AGC) target setting of 2e^5^ (MS and MS/MS).

### Mass spectrometry data analysis

Raw files were searched with the integrated Andromeda search engine in MaxQuant (version 2.0.1.0) against a reviewed human UniProtKB database (release 2020_04), complemented with the default MaxQuant contaminant list. Enzyme specificity was set to trypsin/P and LysC/P. A minimum of 1 unique peptide was used for identification and the default unique + razor for quantification. The match between runs feature was used along with label free quantification with default settings. Cysteine carbamidomethylation was set as a fixed modification and n-terminal acetylation, Methionine oxidation, Asparagine deamination and hydroxyproline were set as variable modifications both for identification and quantification. The false discovery rate (FDR) was set to 1% on both the peptide and protein level. The mass spectrometry data have been deposited to the ProteomeXcange Consortium via the PRIDE partner repository^23^ with the dataset identifier PXD034531.

Heavy and light label free quantification (LFQ)-intensities^24^ were used in the downstream analysis as well as normalized ratio values. The heavy signal originates from proteins produced by the repopulating AEC2, while the light signal comes from the DLS. LFQ-intensity values with MaxQuant defined contaminants removed were normalized across samples by scaling the individual protein intensity values to give the same summed intensity values for all samples. One sample (COPD day 7) was excluded from further analysis as it had only ~ 1% of the average number of detected proteins for the whole dataset. Differential protein expression (DPE) analysis was performed using the DEqMS^25^-application in R, after removing proteins identified in less than half of the 71 samples. All presented p-values are after Benjamini–Hochberg method correction for multiple comparisons. The design matrix was identical to that used in the DESeq2 analysis of RNA-data.

### Histology, immunohistochemistry and immunofluorescence

Cultured lung slices and cell-free control slices were fixated in 4% formaldehyde for 1 h at day 13 of culture. Samples were dehydrated in increasing concentrations of ethanol and Xylen, embedded in paraffin and sectioned at 4 µm. Heat mediated antigen retrieval in a citrate buffer at pH 6 or in a Tris–EDTA buffer at pH 9 using a PT Link (Agilent technologies) or enzymatic antigen retrieval using a commercial pepsin solution (Digest-All 3, Thermo Fisher Scientific) was performed before immunofluorescence (IF) or immunohistochemical (IHC) staining. IHC staining was performed using Dako EnVision Dual Link System-HRP with a 3,3’-Diaminobensidin (DAB) substrate (Agilent technologies) or ImmPRESS AP Horse Anti-Rabbit IgG Polymer Detection Kit, Alkaline Phosphatase with ImmPACT Vector Red Substrate (Vector Laboratories, Burlingame, CA, USA). Antibodies used for IHC and IF are detailed in Supplementary Table 3. Images of entire stained sections were obtained with a VS120 virtual microscopy slide scanning system (Olympus, Tokyo, Japan) and analyzed in QuPath^26^ (version 0.3.0).

### Ethics approval and consent to participate

Informed written consent was obtained from all patients. The use of human material in this study was approved by the Swedish ethical committees in Lund and Gothenburg (Dnr. 2006/91, 2008/413, 20,015/891 and 2016/2, 2018/869), all experimental procedures were performed in accordance with the approved guidelines of the ethical committee.

## Results

**Figure 1:**
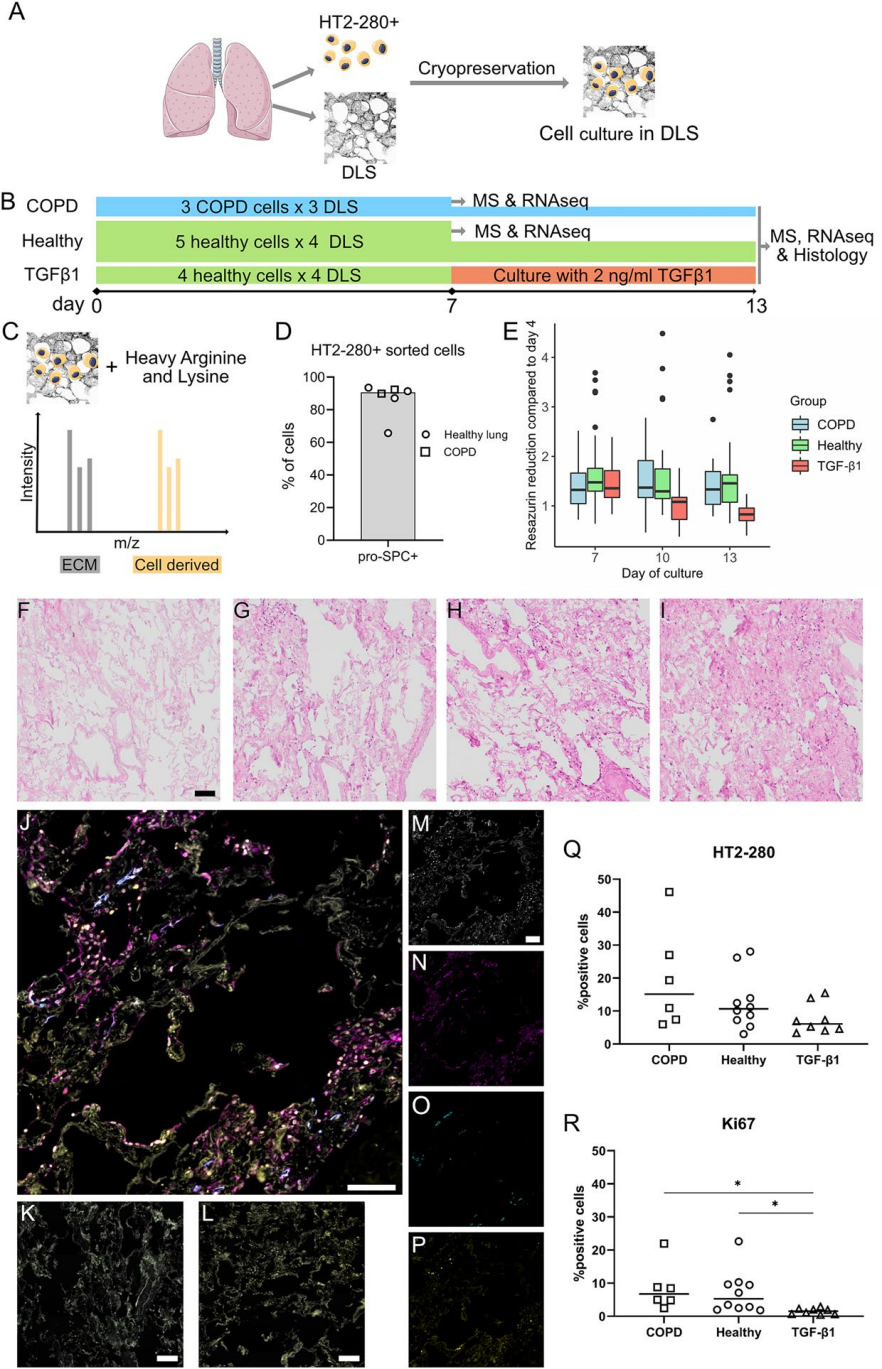
Experimental overview and tissue repopulation of decellularized human lung slices with AEC2 cells. HT2-280 positive cells and tissue for decellularized lung slices (DLS) were isolated from human lungs and cryopreserved until use in tissue culture (**A**). Cell culture in DLS lasted for 13 days, samples with COPD and healthy lung (HL) derived cells were collected at day 7 and day 13, TGF-β1 treated samples at day 13 (**B**), the width of the horizontal bar is proportional to the n for cells. Culture was performed with amino acid labeled with stable heavy isotopes, enabling separate detection of cell derived (yellow) and DLS-derived (gray) proteins in MS analysis, a schematic illustration of peptide peak separation is shown in **C**. Purity of isolated HT2-280 positive cells after cryopreservation evaluated by flow cytometry for the AEC2 marker pro-SPC (**D**). Summary of resazurin measurements of metabolic activity during culture in DLS (**E**). Representative images of H&E stained lung slices after 13 days of culture (**F–I**), from left to right: unseeded control DLS, COPD, HL, and TGF-β1. Immunofluorescence staining (IF) of a lung slice after 13 days of culture (**J**), an unseeded control DLS (**K**) and a seeded negative control where primary antibodies were omitted (**L**). The four channels from (**J**) are shown separately with cell nuclei ((**M**), white), pan-Cytokeratin ((**N**), magenta), HT2-280 ((**O**), Cyan) and Ki-67 ((**P**), yellow). Quantitative analysis of IF staining for HT2-280 (**Q**) and Ki-67 (**R**), statistical testing was performed using pairwise Wilcoxon Rank Sum Tests, each data point represents a unique DLS-cell combination, primary cell numbers for COPD: n = 3, HL: n = 5 and TGF-β1: n = 4. All scale bars are 100 µm.

An overview of the experimental procedures is presented in Fig. 1A–C. In summary, HT2-280 positive cells and tissue for decellularized lung slices (DLS) were isolated from human lungs and cryopreserved until use in tissue culture (Fig. 1A). Cells were cultured in DLS for 13 days. Samples from COPD and healthy lung (HL) derived cells were collected at day 7 and day 13. TGF-β1 (2 ng/mL) was added to HL derived cells at day 7 and samples were collected at day 13. Samples were further processed for either RNA sequencing (RNAseq) and mass spectrometry analysis (MS) or histology (Fig. 1B). Culture was performed with stable isotope labelling by amino acids in cell culture (SILAC), enabling separate detection of newly synthesised proteins (AEC derived proteins, labelled with stable heavy isotopes) and pre-existing, DLS derived ECM proteins (non-labelled) in MS analysis (Fig. 1C).

### Culture in decellularized lung slices support the expansion of cryopreserved AEC2

The AEC2 identity of cryopreserved HT2-280 positive cells were confirmed by flow cytometry analysis of the AEC2 specific marker pro-SPC, revealing that 91% (median) of cells expressed pro-SPC (Fig. 1D and Supplementary Fig. 1). Resazurin reduction by repopulating cells was used as an indirect measurement of cell viability and proliferation after culturing in DLS. This metabolic activity increased significantly between day 4 and 7 of culture, without significant difference between the COPD and Healthy cell groups (Fig. 1E, Supplementary Fig. 2). After the addition of 2 ng/mL of TGF-β1 (day 10 and 13) the metabolic activity in the TGF-ß1 group dropped compared to the day 7 levels and compared to the other groups at the later time points. This could at least in part be explained by an anti-proliferative effect of the added TGF-β1 as IF-evaluation of the proliferation marker Ki-67 revealed lower levels in the TGF-β1 group at day 13 (Fig. 1R). The presence of proliferating cells in the non-treated groups with a stable metabolic activity indicate an active cell turnover maintaining stable cell numbers, while the lowered metabolic levels in the TGF-β1 group might indicate a gradual decline in cell numbers as cells lost e.g. by apoptosis, are replaced at a lower rate. The results from the linear mixed effects model used to evaluate the metabolism assay, showed that 61% of the observed variation came from the difference in DLS donors (data not shown), where a higher metabolic activity was seen in lung slices with a larger surface area after decellularization (data not shown). Only a minor part of the variation was attributable to the cell donor. Presented data on statistically significant group differences in this study are adjusted for DLS effects.

Histological evaluation of repopulated DLS showed that the cells were distributed throughout most of the lung slices (Fig. 1F–I, Supplementary Fig. 3), growing attached to the underlying ECM. Smaller groups of cells were visible, likely reflecting local cell expansion as well as areas where the cells exhibit a morphology consistent with the formation of continuous epithelial layers (Fig. 1G–P).

### Repopulating AEC present a stable expression of AEC markers after an initial proliferative phase

**Figure 2:**
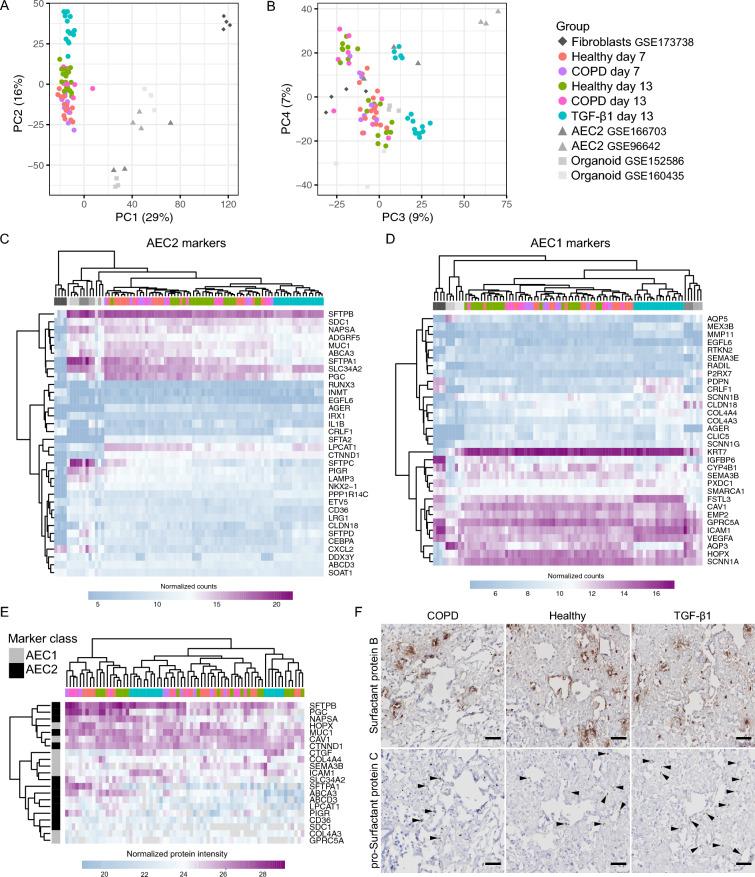
Expression of AEC differentiation markers in cultured HT2-280 positive cells. Principal components analysis comparing transcriptomic profile of the cultured cells with human lung fibroblasts (n = 4, GSE173738), freshly isolated HT2-280 positive AEC2 cells (n = 6, GSE166703 and GSE96642) and AEC2 organoid cultures (GSE152586 and GSE160435) (**A**) and (**B**). Heatmaps of normalized counts for AEC2 (**C**) and AEC1 (**D**) markers from PanglaoDB with included AEC1 marker Caveolin-1 (CAV-1) . Proteomics data for cell derived, heavy isotope labeled AEC1 and AEC2 markers present in > 50% of samples (**E**). Representative images from IHC staining for surfactant protein B (pro- and mature form of the protein) and pro-SPC after 13 days of culture (**F**). Arrowheads mark pro-SPC positive cells, scale bars are 50 µm, positive and negative control stainings can be found in Supplementary Fig. 5.

Evaluation of the percentage of cells staining positive for the AEC2 specific marker HT2-280 after 13 days of culture in DLS showed highly varied expression (3–46% positive cells) with most cells not showing a clear expression of HT2-280 by this method (Fig. 1J,O,Q). However, transcriptomics data showed that both the healthy and COPD-derived cells were more similar to freshly isolated AEC2 (GSE166703 and GSE96642) and AEC2 organoids (GSE152886 and GSE160435) than to lung fibroblasts from the same healthy donors as used in this study (GSE173738), (Fig. 2A and B). The cells of this study showed a degree of dissimilarity to the uncultured AECII similar to that seen in between the reference datasets. As the reference transcriptomes are from freshly isolated cells, part of the global transcriptomic differences observed could possibly be attributed to the effect of in vitro culture. TGF-β1 treatment and, to a lesser extent, time in culture (7 vs. 13 days) made the expression profile of the cultured cells slightly more fibroblast like.

Looking more specifically on known AEC2 (Fig. 2C) and AEC1 (Fig. 2D) markers found in the PanglaoDB^27^, all groups in the current study as expected clustered more closely to the AEC2 reference samples than the fibroblasts. No systematic differences were seen between Healthy and COPD derived cells, and the marker expression also appeared stable over time, while TGF-β1 treated healthy cells clustered on their own. All groups showed expression of the genes for surfactant protein A1, B, C and D, with expression levels of the cultured cells being higher than in the fibroblasts but below or similar to what was seen for the freshly isolated AEC2. The TGF-β1 group had generally lower levels of the surfactant genes and showed an upregulation of some AEC1 genes such as podoplanin (PDPN).

Further evaluation of the AEC phenotype by examining the subset of markers also available in the proteomic data of cell derived proteins in large confirmed the picture revealed by the sequencing data, with no clear trend over time nor difference between the Healthy and COPD group. Interestingly, the transcriptomic alterations induced by TGF-β1 was less evident on the protein level as this group did not clearly cluster by itself, and the only reliably detected surfactant protein, surfactant protein B, showed similar levels across all experimental groups (Fig. 2E). IHC evaluation of surfactant protein B, using an antibody recognizing both the mature protein and the pro-form, showed positive staining in and close to a fraction of the cells (Fig. 2F). No surfactant protein B staining was observed in cell-free control DLS incubated in medium for 13 days (Supplementary Fig. 5), indicating an active production and secretion of the protein from AEC in repopulated DLS. A smaller proportion of the cells showed positive IHC staining for pro-SPC after 13 days of culture (Fig. 2F), indicating that most of the cells had lost this AEC2 specific marker which, according to flow cytometry data (Fig. 1D), was present in about 90% of the cells before seeding in DLS. However, the frequency of pro-SPC positive cells appeared somewhat similar to that seen among the AEC in native healthy lung with the same staining (Supplementary Fig. 5). To visualize whether some of the AEC2 had differentiated to epithelial cells with a AEC1-like phenotype after 13 days in culture, caveolin-1 (CAV1) was used as a marker of AEC1 in combination with pan-cytokeratin (Supplementary Fig. 6A–E). Our IHC stainings showed a mixed AEC population in terms of morphology, where many of the cells still presented a cuboidal AEC2 morphology and some cells had gained a squamous cell shape, typical of AEC1. CAV1 was detected in cells with squamous morphology corresponding to an AEC1 phenotype, but also in cells with a cuboidal morphology, which may suggest that some of the cells are in an intermediate/transitioning state between an AEC2 and AEC1 phenotype (. In line with these observations, CAV1 expression was also detected in the DLS cultures by RNAseq and MS analysis (Fig. 2). These data confirm the matrisome profile that there is a mixed AEC population after 13 days in culture.

Although the TGF-β1 group had a marker expression distinct from that of fibroblasts and maintained a high expression of epithelial keratin 7 (Fig. 2D), the altered marker expression could in part be related to a TGF-β1 induced change towards a mesenchymal phenotype. In support of such a change we observed a relative upregulation of classical EMT related genes such as VIM and FN1 (Supplementary Fig. 4). However, IHC stainings for the mesenchymal markers CD90 and platelet derived growth factor receptor α (PDGFR-α) did not reveal any positive cells (Supplementary Fig. 7). These stainings also serve as controls for the potential presence of contaminating mesenchymal cells in the cultures. The stainings together with the overall gene expression profiles confirm that the studied cells in the DLS are of an epithelial phenotype with a AEC2 origin.

### AEC are competent matrisome producing cells

The matrisome classification created by Naba et al. is a helpful tool for understanding and organizing complex alterations in ECM composition, and the result of this study is partially structured around this classification^28^. It divides proteins into core matrisome proteins and ECM-associated proteins. Core matrisome include collagens, ECM glycoproteins and proteoglycans. ECM-associated proteins include ECM-affiliated proteins, ECM regulators and secreted factors.

**Figure 3:**
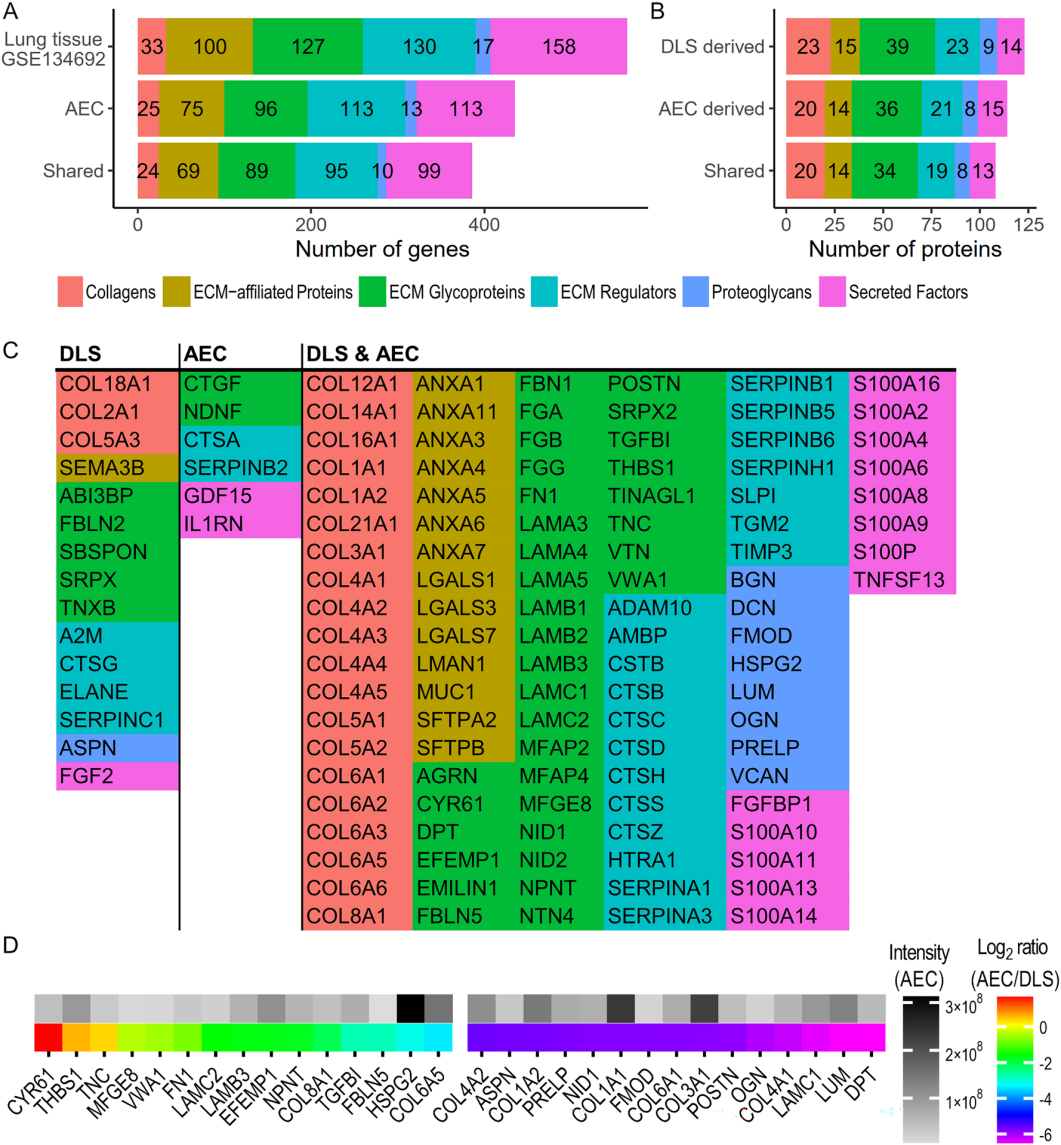
Matrisome production of AEC derived from healthy lungs. Number of genes belonging to the different matrisome groups expressed at culture day 13 compared to transcriptomic data from whole normal lung from the previously published dataset GSE134692 (**A**), included genes were present in > 75% of all samples in the respective datasets. Number of detected matrisome proteins from the decellularized lung ECM in the DLS compared to what are produced by the repopulating AEC cells (**B**), included proteins were detected in > 75% of healthy day 13 samples. Gene names of proteins from (**B**) detected uniquely from the DLS or from the repopulating cells or in both, grouped into their respective matrisome group (**C**). MS data of core matrisome proteins detected from both DLS (non-labelled proteins) and produced by AEC (heavy isotope labelled) at culture day 13 (**D**). The 15 proteins with the highest and lowest median ratios (new/old) are displayed (gene names) beside their normalized median intensity value.

A comparison of matrisome genes expressed in the healthy group at day 13 in this study with another RNA-sequencing dataset from peripheral lung homogenate showed that AEC expressed the majority of matrisome genes found in whole peripheral lung (Fig. 3A). Focusing on the largely structural core matrisome, AEC expressed 123/177 core matrisome genes found in the reference dataset, and an additional 10 not found there. One collagen gene, COL17A1, found uniquely in AEC (Supplementary Table 4) is a transmembrane hemidesmosome component, found to be important for maintaining AEC2 stem cell function in mice^29^. Other genes uniquely expressed in AEC might more reflect the process of re-epithelialization, such as FGA and FGG (Fig. 3A, Supplementary Table 4), coding for fibrinogen chains, which is deposited into ECM by AEC and upregulated during epithelial healing^30,31^. Many BM constituents were among the genes common between the two datasets, but also genes for fibrillar collagens such as collagen type I, II, III and V (Supplementary Table 4).

The isotope labelling made it possible for the MS analysis to provide data on both the old ECM in the DLS and the newly produced proteins. The repopulating AEC had at day 13, produced 114 different matrisome proteins as compared to 123 detected from the decellularized lung ECM, where 108 were shared between them (Fig. 3B). In the whole set of core matrisome proteins detected from the healthy AEC day 13 in the model, it is noteworthy that the cells produced 20 different collagen chains of 10 different collagens as well as all of the laminin chains prominent in adult lung tissue i.e. laminin α3-5, β1-3 and γ1 and γ2^32,33^ (Fig. 3C). The normalized ratio values (heavy cell derived / light DLS derived) provided by the MaxQuant analysis gave a picture of how the AEC matrisome production relates to the composition of the pre-existing ECM. For the core matrisome proteins which make up the bulk of the decellularized lung tissue the ratio of new/old was < 1. The 30 core matrisome proteins with the highest and lowest ratios are summarized in Fig. 3D (Supplementary Table 5 lists all core matrisome proteins with a ratio). Among the proteins with a high ratio, several were related to cell adhesion, such as tenascin (TNC) and fibronectin (FN1) as well as BM constituent such as laminins and collagen 8 (COL8A1). The latter is a prominent constituent of vascular BMs, of relevance as AEC partly share BMs with endothelial cell in alveoli. Among the protein with the lowest ratio, collagen type 1 and 3 chains stand out as they displayed a high heavy (new) protein intensity, indicative of substantial production, but the ratio remained low as they constitute a significant proportion of the decellularized ECM.

### Minor differences between AEC2 from end stage COPD and healthy lungs grown in healthy ECM

**Figure 4:**
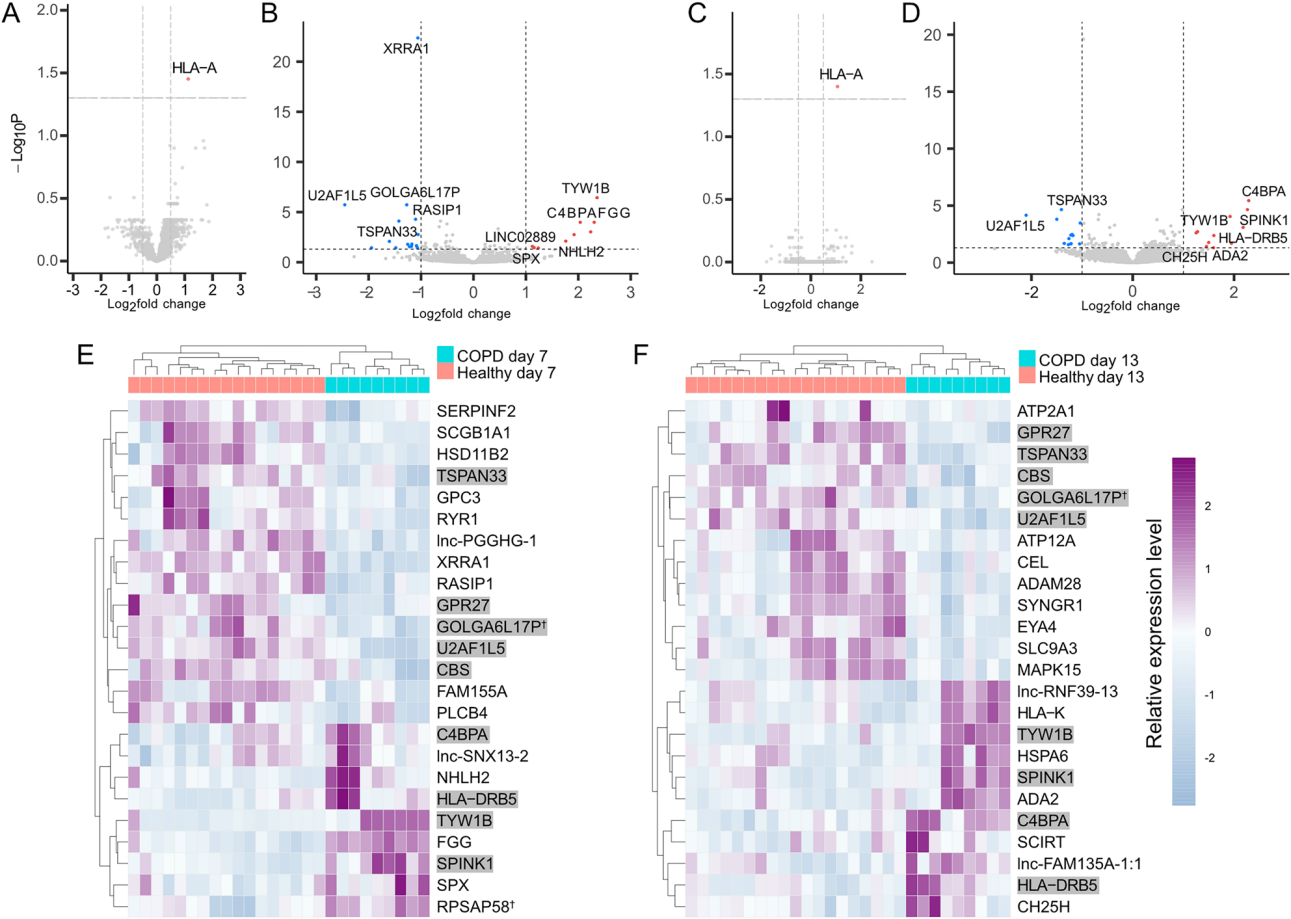
COPD related changes in gene and protein expression. Volcano plots of differentially expressed proteins and genes after 7 (**A**) and (**B**) and 13 days (**C**) and (**D**) of culture in DLS. Analysis was done with DEqMS for MS-data and DESeq2 for RNA-sequencing data. Red means upregulated in COPD, blue means downregulated, cutoffs are set at an adjusted p-value < 0,05 and fold change > log2(1,5). Heatmaps of normalized count data for the differentially expressed genes from B and D (**E**) and (**F**), genes common between the two time points are shaded in gray. Genes marked with a cross are considered pseudogenes, each heatmap column represents a unique DLS-cell combination, cell n = 5 for untreated and n = 3 for the COPD group.

MS-data showed only one differing protein between the COPD and healthy group, HLA class I histocompatibility antigen coded by the gene HLA-A. This protein was upregulated in COPD at both time points, exhibiting slightly more than double the normalized intensity values of the healthy group (Fig. 4A and C). RNA-sequencing did yield a larger number of differing genes, 24 at each time point, a handful of which were long non-coding RNAs or pseudogenes (Fig. 4B and D–F). Nine of the genes (TSPAN33, GPR27, CBS, GOLGA6LP17, U2AF1L5, C4BPA, HLA-DRB5, TYW1B and SPINK1) were common between the time points 7-days and 13-days and might as such be of greater mechanistic significance (Fig. 4E and F).

### AEC exhibits extensive plasticity in gene expression in response to TGF-β1

**Figure 5:**
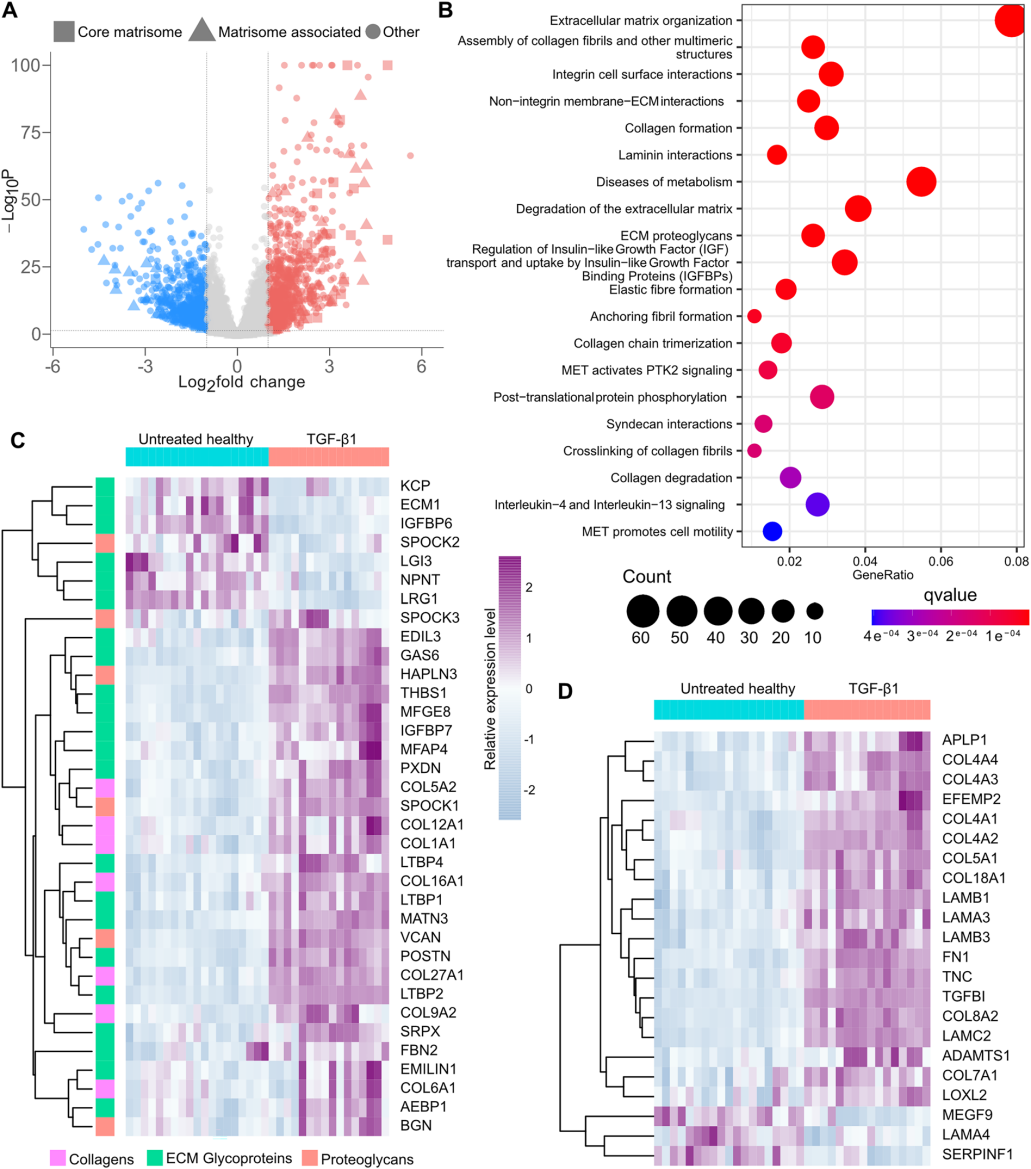
TGF-β1 induced gene expression changes in healthy lung-derived AEC. 1418 genes were found to have a significantly altered expression defined as adjusted p-value < 0,05 and log2 fold change > 1 (**A**). Top 20 pathways most significantly over-represented in Reactome pathway analysis of the differentially expressed genes (**B**). Heatmaps of normalized counts for differentially expressed core matrisome genes (**C**) and (**D**), separated according to if they have a basement membrane GO-component annotation, with non-BM genes in (**C**) and basement membrane genes in (**D**). Each heatmap column represents a unique DLS-cell combination, cell n = 5 for untreated and n = 4 for the TGF-β1 group.

Healthy AEC exposed to TGF-β1 from day 7 to 13 in culture had differently expressed genes with a log2 fold change > 1, many of the genes with the largest increase in expression were matrisome proteins (Fig. 5A). In line with this, pathway analysis with the differentially expressed genes using the Reactome database resulted in several ECM-turnover related pathways among the most significantly enriched (Fig. 5B). Heatmaps with differentially expressed core matrisome genes divided by their association to BM according to GO-compartment annotation (Fig. 5C and D). With the TGF-β1 stimuli, most of the differentially expressed core matrisome genes showed upregulation, this includes genes for several fibrillar and fibrillar associated collagens such as type I, V, XXVII, XII and XVI (Fig. 5C). Also, many BM collagens are upregulated including four of the six known collagen type IV sub-chains (Fig. 5D). Among the laminin genes, four are upregulated and one is downregulated, altering the potential laminin trimers forming in the BM. Three of the upregulated laminins make up the constituents of laminin 332 (formerly laminin-5), which is expressed by regenerating epithelium in lungs affected by idiopathic pulmonary fibrosis (IPF) and cryptogenic organizing pneumonia (COP)^34^. Laminin-332 is well known to bind to the homotrimeric collagen type VII, which is also among the upregulated BM genes in the dataset.

Several genes within the matrisome associated group showed a clear alteration in expression pattern after TGF-β1 stimuli (Supplementary Fig. 8). The surfactant expression profile showed lowered expression for surfactant -A1, -A2, -A3 and -C, with a simultaneous increase for surfactant protein D. Within the ECM affiliated group, an altered cell signalling profile was apparent with a switch in the expression of several semaphorins and glypican genes. ECM regulators showed a complex change in expression profile with several ECM modifying proteases and protease inhibitors being upregulated while others had a decreased expression. In the final matrisome group, the secreted factors, regulation of several pathways was apparent, among which the Wnt family of pathways was prominent. In addition, several leukocyte attracting chemokines showed upregulation after TGF-β1 stimulation (Supplementary Fig. 8). A complete list of upregulated genes can be found in Supplementary Table 6.

### TGF-ß1 stimulated AEC upregulate matrisome and cell adhesion proteins

**Figure 6:**
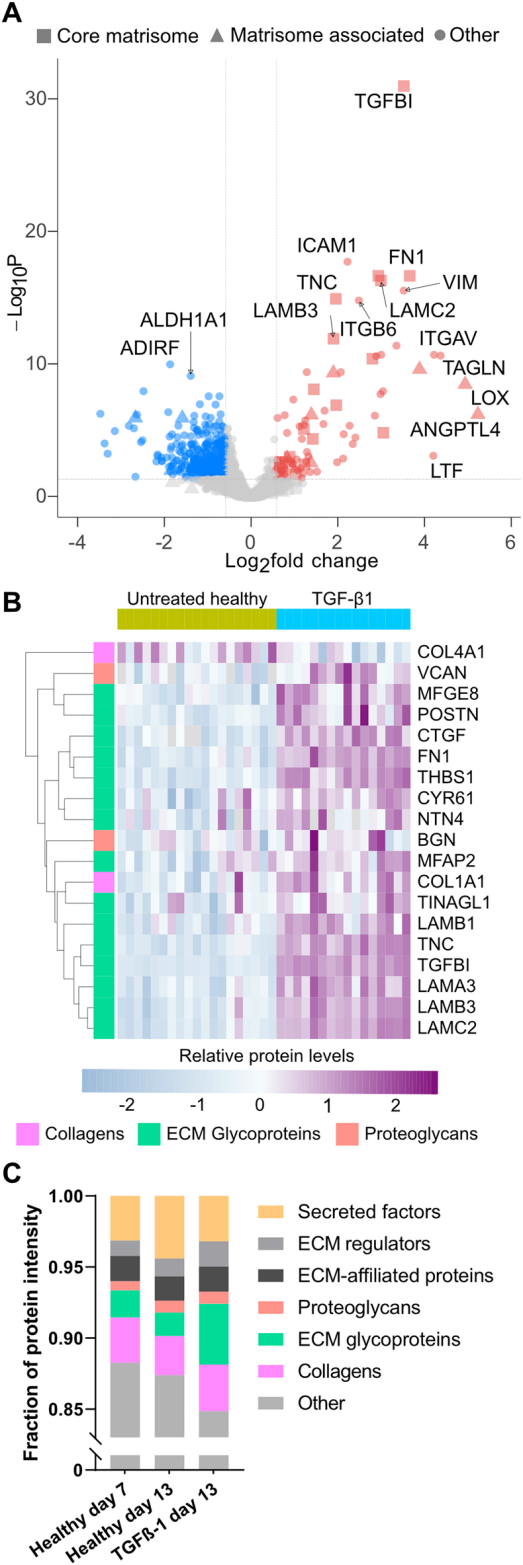
TGF-β1 induced protein expression changes in AEC. Volcano plot of results from DEqMS-analysis comparing TGF-β1 treated cells with untreated cells (**A**), red indicates upregulated in response to TGF-β1, blue indicates downregulated. Heatmap of normalized intensity values for differentially expressed core matrisome proteins (**B**), each column represents a unique DLS-cell combination, cell n = 5 for untreated and n = 4 for the TGF-β1 group. The proportion of matrisome proteins produced by the AEC was estimated by summing the normalized intensity signals within each group (**C**).

Matrisome proteins dominate among the most upregulated proteins in response to TGF-β1, to an even larger extent than for the gene expression data (Fig. 6A, Supplementary Table 7). Among the upregulated proteins several proteins important for cell adhesion were found, such as integrins, intercellular adhesion molecule 1 (ICAM1), transforming growth factor-beta-induced protein ig-h3 (TGFBI), fibronectin (FN1) and tenascin C (TNC). For the evaluation of this system as a model to study lung fibrosis, it is of note that the TGF-β1 treatment also led to upregulation of periostin (POSTN) and versican (VCAN), which together with fibronectin and Tenascin C have been shown to be upregulated in fibroblastic foci, which are considered to constitute remodelling hotspots in IPF^35,36^. As might be expected, several of the highly upregulated proteins are known downstream targets of TGF-β1 such as CYR61, CTGF and TGFBI. Focusing on core matrisome proteins, the laminins match the pattern of upregulation from the RNA-sequencing data (Fig. 6B) while others like the alpha-1 chain of collagen type IV show signs of posttranscriptional regulation based on the downregulated protein expression despite upregulation in the RNA data. Noteworthy is also that upregulation of the enzyme lysyl oxidase (LOX) was detected, which contributes to ECM crosslinking.

Among the differentially expressed matrisome associated proteins (Supplementary Fig. 9), several proteins that may influence the ECM turnover in the lung, e.g. alpha-1 antitrypsin (SERPINA1) and alpha 1-antichymotrypsin (SERPINA3) were upregulated while fibroblast growth factor binding protein 1 (FGFBP1) and metalloproteinase inhibitor 3 (TIMP3) were downregulated. Finally, looking at the summed normalized intensity within the different groups, a trend towards an increasing matrisome proportion out of the new protein was observed from day 7 to day 13, consistent with extracellular accumulation over time (Fig. 6C). TGF-β1 treatment increased the proportion of matrisome proteins even more, mainly due to larger accumulation of ECM glycoproteins and ECM regulators.

## Discussion

Our results show that primary human AEC2 cultured in a human lung ECM environment maintains expression of important AEC markers and that the cells produce a broad and complex set of ECM proteins. The ECM production capabilities stretch well beyond basement membrane constituent expected by any epithelial cell, and include structural proteins found outside the BM. AEC2 from end stage COPD patients showed unexpected similarity to the healthy control cells, potentially related to the healthy ECM culture substrate. Finally, the plasticity in ECM production when exposed to an external profibrotic stimulus in the form of TGF-β1 supports the notion that AEC2 can take active part in lung ECM turnover, especially in fibrotic remodelling in the human lung.

During recent years, gene expression studies have been made to further understand and characterize the AEC in health and disease using single cell analysis and RNA-sequencing. Negretti et al. has performed a single-cell atlas of the developing mouse lung where they show an important role of AEC1 in the expression of genes associated with elastin structures and the supporting alveolar ECM during organogenesis^37^ and Zepp et al. generated another single-cell RNA-sequencing atlas of the developing murine lung during alveologenesis where they show that paracrine signalling from AECI are important for alveolar formation^38^. The IPF Cell Atlas which contains human cells from IPF and healthy patients^39,40^ are an important and useful single-cell atlas generated from RNA-sequencing of IPF lungs, healthy donor lungs and also COPD lungs focusing on aberrant epithelial, fibroblast, and endothelial cell populations^39^. In the current study we explored the plasticity of AEC2 cells to actively be involved in ongoing remodelling processes by characterizing the entire matrisome generated by the AEC2 on both gene and protein level to fully uncover the potential of these cells which has not previously been shown.

The cultured AEC2 retained markers of AEC2 after 7 and 13 days of culture, but as only a minority of the cultured cells were positive for the HT2-280 marker in IF staining, it is likely that a proportion have changed their phenotype, where a AEC1 like phenotype could be expected^41,42^ suggesting an intermediate epithelial cell population which was confirmed by the obtained matrisome profile and IHC stainings. It has previously been demonstrated with single cell RNA sequencing a subpopulation of cells that expressed features of both AEC2 and AEC1 cells in both control and IPF lungs^40,43^, where Habermann et al. at gene level identified novel epithelial cell phenotypes, including a *KRT5*^*−*^/*KRT17*^+^ ECM-producing epithelial cell population in the lungs obtained from patients with pulmonary fibrosis^40^.

The limited staining expression of HT2-280 and pro-SPC in AEC culture suggest that the culture has influenced the expression of these markers and that they may have lost some of their AEC2 identity and potentially gained AEC1 identity. However, out of the AEC1 and AEC2 markers investigated by RNAseq, AEC cultures show a stable expression of a great majority of AEC2 markers over time, including surfactant proteins, suggesting that their phenotype is maintained between the time points investigated in the present study. Importantly, the AEC cultures showed a similar expression pattern of AEC1 and AEC2 markers compared to fresh AEC2 (reference samples), with similar expression levels in contrast to fibroblast control samples. However, a direct statistical comparison between the reference sample and the AEC cultures was not suitable as data is derived from different studies.

Our data indicate that AEC1 markers increased within TGF-β1 treated cells, and in line with AEC differentiation these cells had a lower proportion of proliferative cells. These findings are in line with known senescence inducing effects of TGF-β1 and AEC2 senescence seen in lung fibrosis^44–46^. Measurements of cell metabolism and Ki67 staining at day 13 indicated that AEC2 proliferated in our culture system, however cell numbers seemed to have reached a steady state by day 7, why our day 13 data might be less influenced by the process of re-epithelialization.

Gene expression data showed that the AEC expressed the majority of core matrisome genes expressed in lung tissue and in addition to the expected BM constituents, a plethora of more interstitial ECM genes often associated with mesenchymal cells were prominent. These findings were confirmed by the proteomics data, that despite a generally lower coverage, picked up a significant production of interstitial ECM proteins such as the fibrillar collagens type I, II, III and V as well as the proteoglycans lumican and asporin. These findings motivate further studies of the role of AEC in ECM maintenance and remodelling of the human lung.

One somewhat surprising finding was that the COPD-derived cells proliferated at least as well as the healthy cells and the differences in gene and protein expression were limited. Other studies, mainly focused on mesenchymal cells, have shown that cells tend to adopt a phenotype matching the surrounding ECM^47,48^. In a previous paper, we compared the behaviour and matrisome production profile of healthy lung fibroblasts cultured in healthy and IPF derived decellularized human lung scaffolds. The IPF scaffolds induced production of disease-associated matrisome proteins from healthy lung fibroblasts^48^. As the lack of a baseline comparison between COPD derived and healthy cells before culture in DLS precludes any conclusion, it remains an intriguing possibility that signals from the healthy-derived ECM might have helped normalize any pathological changes in AEC2 of COPD patients. Further studies evaluating AEC2 in COPD-ECM environment would be of value to further validate this notion. The only COPD related statistically significant difference in the MS analysis was an upregulation of HLA-A. Increased HLA-A protein have previously been found in exhaled breath condensate of COPD patients, another study found increased gene expression in both AEC2 and in blood samples of COPD patients^49,50^. Our results both support HLA-A as a potential COPD biomarker and indicate that the AEC2 retain characteristics of their origin in this culture system. As HLA-A presents intracellular antigens to immune cells e.g., viral antigens, it is of interest that the cystathionine β-synthase (CBS) gene was downregulated in the COPD group. This enzyme catalyzes the production of the gasotransmitter hydrogen sulfide, which functions in the immune responses to viral infection^51^. Together with the HLA-A finding this could indicate an altered response to viral infections, which could be part of the pathogenesis of virus-induced COPD exacerbations^52^. Adams et al. also observed minor changes at gene level between COPD and control lungs which may depend on end stage lungs, whereas there were large differences between IPF and control lungs in their gene data base^39^.

Healthy derived AEC2 did in our study show a response to TGF-β1 in many respects similar to what have been observed for fibroblasts in vitro, with a gene expression profile compatible with altered turnover and increased crosslinking of ECM, and increased ECM protein expression^53^. Also, the MS-results show increased ECM deposition with specific changes in basement membrane composition. Increased expression of the three chains of laminin-332, which are upregulated in the epithelium of bronchiolised distal air spaces in IPF, is of particular interest as also the gene for collagen type VII is upregulated^34^. Upregulation of the collagen type VII gene was previously found in a study of idiopathic interstitial pneumonias (IIPs), where 7/12 patients were diagnosed with IPF^54^. Laminin-332 binds to collagen type VII and is important for cell adhesion and promotes cancer cell migration^55^. Our findings support the relevance of the presented model system and the proposition by Lappi-Blanco et.al., that the regenerating epithelium of bronchiolised air spaces of IPF patients are of AEC2 origin, as opposed to bronchial epithelial cells^34^. Two markers of bronchiolised airspaces have been established, SOX2 and MUC5B, and transdifferentiated basal cells have been suggested as an alternative source of these cells, partly based on the high expression of MUC5B in airway submucosal glands^56^. The AEC in our dataset do express both markers, but the expression levels decrease with TGF-β1 exposure, which might speak against AEC source of the epithelium of bronchiolised airspaces or indicating that another epithelial subcell type could be involved in this process^39^.

In this study we used healthy decellularized lung tissue slices as a 3D in vitro culture model for AEC2. The motivation for using the DLS was to maintain as much as possible of native tissue properties, including biomechanical properties that better resemble native tissue than traditional tissue culture plastic. Balancing the efficient removal of cells while at the same time preserving ECM composition and structure is important to achieve a physiological culture model and avoid artefacts and ECM degradation. We have previously shown with histological analysis the preservation of overall tissue morphology, elastic fibres and basement membrane collagen in the lung slices after the decellularization process^10^. A future approach would be to culture healthy and diseased AEC2 on COPD or IPF-derived DLS to to investigate and compare the AEC2 phenotype and matrisome profile over time. Substrate stiffness is an important aspect of cell culture systems and can greatly affect cell behaviour. The DLS provide a significantly more compliant culture substrate than stiff tissue culture plastic by providing a relevant ECM milieu for the study of lung cells. Although a somewhat increased stiffness was observed in the healthy DLS compared to native healthy lung slices, the healthy DLS still showed a reduced stiffness in comparison to the IPF derived DLS which had an increased stiffness more similar to the native IPF tissue^48^. The repopulated DLS used in the current study were kept as free-floating scaffolds and were not attached to any other material in order to reduce the resistance and “transfer” of stiffness to the DLS. The goal of the present study was to investigate if it was possible to culture the primary AEC2 in the DSLs and maintain their phenotype while following ECM synthesis with and without profibrotic stimuli using SILAC. A further goal is to study the effect of mechanical forces mimicking breathing patterns on the AEC2 capacity and plasticity by using a newly developed culture system applying physiomimetic layers^57^ that may further improve the relevance of in vitro results to in vivo conditions.

One limitation in this study is the sex profile of the donors, with only one cell donor in each group being female, and none of the DLS coming from a female donor. No obvious sex related patterns were found, but the male predominance should be kept in mind when interpreting the data. A strength of the study is the use of multiple DLS donors in combination with multiple cell donors, especially for the healthy cells, and the results from the differential expression analysis should thereby be relatively robust.

## Conclusions

This study introduces a novel model system with cryopreserved AEC2 cultured in a human ECM environment without prior expansion in 2D culture, thereby preserving phenotypic traits of the in vivo origin. Results from RNA-seq and MS, i.e. both gene and protein level replicate known disease related alterations both in COPD cells and healthy cells in a pro-fibrotic environment, induced by TGF-β1. Finally, a previously unexplored capacity of AEC2 to directly contribute to ECM remodelling was revealed, motivating further studies of the contribution of AEC to remodelling events in chronic lung disease. These datasets can be valuable resources for future studies of AEC, as the amount of publicly available combined sequencing and proteomics data is currently limited.

## Supplementary Information

Supplementary Tables.

Supplementary Figures.

## Data Availability

The datasets generated during the current study are available through the ProteomeXcange Consortium, dataset identifier PXD034531, and the NCBI's Gene Expression Omnibus, GEO Series accession number GSE191279. R scripts are provided in the supplementary files. Other generated data and relevant files are available from the corresponding author on reasonable request.

## Abbreviations

AEC1: Airway epithelial cell type I
AEC2: Airway epithelial cell type II
SERPINA1: Alpha-1 antitrypsin
SERPINA3: Alpha 1-antichymotrypsin
BM: Basement membrane
CBS: Cystathionine β-synthase
COPD: Chronic obstructive pulmonary disease
DLS: Decellularized lung slices
ECM: Extracellular matrix
FGFBP1: Fibroblast growth factor binding protein 1
FN1: Fibronectin 1
HL: Healthy lung
ICAM1: Intercellular adhesion molecule 1
IIP: Idiopathic interstitial pneumonias
IPF: Idiopathic pulmonary fibrosis
MS: Mass spectrometry
RNAseq: RNA sequencing
TNC: Tenascin C
TGF-ß1: Transforming growth factor ß1
TIMP3: Metalloproteinase inhibitor 3

## References

[1] Burgstaller G, Oehrle B, Gerckens M, White ES, Schiller HB, Eickelberg O (2017). The instructive extracellular matrix of the lung: Basic composition and alterations in chronic lung disease. Eur. Respir. J..

[2] Burgess JK, Harmsen MC (2022). Chronic lung diseases: Entangled in extracellular matrix. Eur. Respir. Rev..

[3] Barkauskas CE, Cronce MJ, Rackley CR (2013). Type 2 alveolar cells are stem cells in adult lung. J. Clin. Invest..

[4] Hinz B, Phan SH, Thannickal VJ (2012). Recent developments in myofibroblast biology: Paradigms for connective tissue remodeling. Am. J. Pathol..

[5] Parimon T, Yao C, Stripp BR, Noble PW, Chen P (2020). Alveolar epithelial type II cells as drivers of lung fibrosis in idiopathic pulmonary fibrosis. Int. J. Mol. Sci..

[6] Tsuji T, Aoshiba K, Nagai A (2006). Alveolar cell senescence in patients with pulmonary emphysema. Am. J. Respir. Crit. Care Med..

[7] Yue X, Shan B, Lasky JA (2010). TGF-β: Titan of lung fibrogenesis. Curr. Enzym. Inhib..

[8] Dobbs LG, Gonzalez RF. Isolation and Culture of Pulmonary Alveolar Epithelial Type II Cells. https://onlinelibrary.wiley.com/doi/pdf/10.1002/0471221201.ch9 Accessed May 4, 2018.

[9] Gonzalez RF, Allen L, Gonzales L, Ballard PL, Dobbs LG (2010). HTII-280, a biomarker specific to the apical plasma membrane of human lung alveolar type II cells. J. Histochem. Cytochem..

[10] Rosmark O, Åhrman E, Müller C (2018). Quantifying extracellular matrix turnover in human lung scaffold cultures. Sci. Rep..

[11] Bates D, Mächler M, Bolker BM, Walker SC (2015). Fitting linear mixed-effects models using lme4. J. Stat. Softw..

[12] Daniel Lüdecke. sjPlot: Data Visualization for Statistics in Social Science. Published online 2020. https://cran.r-project.org/package=sjPlot

[13] Edgar R, Domrachev M, Lash AE (2002). Gene expression omnibus: NCBI gene expression and hybridization array data repository. Nucleic Acids Res..

[14] Love MI, Huber W, Anders S (2014). Moderated estimation of fold change and dispersion for RNA-seq data with DESeq2. Genome Biol..

[15] Bolger AM, Lohse M, Usadel B (2014). Trimmomatic: A flexible trimmer for Illumina sequence data. Bioinformatics.

[16] Dobin A, Davis CA, Schlesinger F (2013). STAR: Ultrafast universal RNA-seq aligner. Bioinformatics.

[17] Kadefors M, Rolandsson Enes S, Åhrman E (2021). CD105+CD90+CD13+ identifies a clonogenic subset of adventitial lung fibroblasts. Sci. Rep..

[18] Jacob A, Morley M, Hawkins F (2017). Differentiation of human pluripotent stem cells into functional lung alveolar epithelial cells. Cell Stem Cell.

[19] Katsura H, Sontake V, Tata A (2020). Human lung stem cell-based alveolospheres provide insights into SARS-CoV-2-mediated interferon responses and pneumocyte dysfunction. Cell Stem Cell.

[20] Mulay A, Konda B, Garcia G (2021). SARS-CoV-2 infection of primary human lung epithelium for COVID-19 modeling and drug discovery. Cell. Rep..

[21] Sivakumar P, Thompson JR, Ammar R (2019). RNA sequencing of transplant-stage idiopathic pulmonary fibrosis lung reveals unique pathway regulation. ERJ Open Res..

[22] Raivo K. pheatmap: Pretty Heatmaps. R package version 1.0.12. Published 2019. https://cran.r-project.org/package=pheatmap.

[23] Perez-Riverol Y, Csordas A, Bai J (2019). The PRIDE database and related tools and resources in 2019: Improving support for quantification data. Nucleic Acids Res..

[24] Cox J, Hein MY, Luber CA, Paron I, Nagaraj N, Mann M (2014). Accurate proteome-wide label-free quantification by delayed normalization and maximal peptide ratio extraction, termed MaxLFQ. Mol. Cell. Proteomics.

[25] Zhu Y, Orre LM, Zhou Tran Y (2020). DEqMS: A method for accurate variance estimation in differential protein expression analysis. Mol. Cell. Proteomics.

[26] Bankhead P, Loughrey MB, Fernández JA (2017). QuPath: Open source software for digital pathology image analysis. Sci. Rep..

[27] Franzén O, Gan LM, Björkegren JLM (2019). PanglaoDB: A web server for exploration of mouse and human single-cell RNA sequencing data. Database.

[28] Naba A, Clauser KR, Hoersch S, Liu H, Carr SA, Hynes RO (2012). The matrisome: in silico definition and in vivo characterization by proteomics of normal and tumor extracellular matrices. Mol. Cell. Proteomics.

[29] Otsubo K, Goto H, Nishio M (2017). MOB1-YAP1/TAZ-NKX2.1 axis controls bronchioalveolar cell differentiation, adhesion and tumour formation. Oncogene.

[30] Guadiz G, Sporn LA, Simpson-Haidaris PJ (1997). Thrombin cleavage-independent deposition of fibrinogen in extracellular matrices. Blood.

[31] Perrio MJ, Ewen D, Trevethick MA, Salmon GP, Shute JK (2007). Fibrin formation by wounded bronchial epithelial cell layers in vitro is essential for normal epithelial repair and independent of plasma proteins. Clin. Exp. Allergy.

[32] Pierce RA, Griffin GL, Mudd MS (1998). Expression of laminin alpha3, alpha4, and alpha5 chains by alveolar epithelial cells and fibroblasts. Am. J. Respir. Cell Mol. Biol..

[33] Pierce RA, Griffin GL, Miner JH, Senior RM (2000). Expression patterns of laminin alpha1 and alpha5 in human lung during development. Am. J. Respir. Cell Mol. Biol..

[34] Lappi-Blanco E, Kaarteenaho-Wiik R, Salo S (2004). Laminin-5 gamma2 chain in cryptogenic organizing pneumonia and idiopathic pulmonary fibrosis. Am. J. Respir. Crit. Care Med..

[35] Wolters PJ, Collard HR, Jones KD (2014). Pathogenesis of idiopathic pulmonary fibrosis. Annu. Rev. Pathol..

[36] Estany S, Vicens-Zygmunt V, Llatjós R (2014). Lung fibrotic tenascin-C upregulation is associated with other extracellular matrix proteins and induced by TGFβ1. BMC Pulm. Med..

[37] Negretti NM, Plosa EJ, Benjamin JT (2021). A single-cell atlas of mouse lung development. Development.

[38] Zepp JA, Morley MP, Loebel C (2021). Genomic, epigenomic, and biophysical cues controlling the emergence of the lung alveolus. Science.

[39] Adams TS, Schupp JC, Poli S (2020). Single-cell RNA-seq reveals ectopic and aberrant lung-resident cell populations in idiopathic pulmonary fibrosis. Sci. Adv..

[40] Habermann AC, Gutierrez AJ, Bui LT (2020). Single-cell RNA sequencing reveals profibrotic roles of distinct epithelial and mesenchymal lineages in pulmonary fibrosis. Sci. Adv..

[41] Liebler JM, Marconett CN, Juul N (2016). Combinations of differentiation markers distinguish subpopulations of alveolar epithelial cells in adult lung. Am. J. Physiol. Lung Cell. Mol. Physiol..

[42] Barkauskas CE, Chung M-I, Fioret B, Gao X, Katsura H, Hogan BLM (2017). Lung organoids: Current uses and future promise. Development.

[43] Xu Y, Mizuno T, Sridharan A (2016). Single-cell RNA sequencing identifies diverse roles of epithelial cells in idiopathic pulmonary fibrosis. JCI Insight.

[44] Minagawa S, Araya J, Numata T (2011). Accelerated epithelial cell senescence in IPF and the inhibitory role of SIRT6 in TGF-β-induced senescence of human bronchial epithelial cells. Am. J. Physiol. Lung Cell. Mol. Physiol..

[45] Katakura Y, Nakata E, Miura T, Shirahata S (1999). Transforming growth factor beta triggers two independent-senescence programs in cancer cells. Biochem. Biophys. Res. Commun..

[46] Chilosi M, Carloni A, Rossi A, Poletti V (2013). Premature lung aging and cellular senescence in the pathogenesis of idiopathic pulmonary fibrosis and COPD/emphysema. Transl. Res..

[47] Parker MW, Rossi D, Peterson M (2014). Fibrotic extracellular matrix activates a profibrotic positive feedback loop. J. Clin. Invest..

[48] Elowsson Rendin L, Löfdahl A, Åhrman E (2019). Matrisome properties of scaffolds direct fibroblasts in idiopathic pulmonary fibrosis. Int. J. Mol. Sci..

[49] Kubysheva N, Soodaeva S, Novikov V (2018). Soluble HLA-I and HLA-II molecules are potential prognostic markers of progression of systemic and local inflammation in patients with COPD. Dis. Markers.

[50] Wei L, Xu D, Qian Y (2015). Comprehensive analysis of gene-expression profile in chronic obstructive pulmonary disease. Int. J. Chron. Obstruct. Pulmon. Dis..

[51] Bazhanov N, Ansar M, Ivanciuc T, Garofalo RP, Casola A (2017). Hydrogen sulfide: A novel player in airway development, pathophysiology of respiratory diseases, and antiviral defenses. Am. J. Respir. Cell Mol. Biol..

[52] Kurai D, Saraya T, Ishii H, Takizawa H (2013). Virus-induced exacerbations in asthma and COPD. Front. Microbiol..

[53] Merl-Pham J, Basak T, Knüppel L (2019). Quantitative proteomic profiling of extracellular matrix and site-specific collagen post-translational modifications in an in vitro model of lung fibrosis. Matrix Biol. Plus.

[54] Horimasu Y, Ishikawa N, Taniwaki M (2017). Gene expression profiling of idiopathic interstitial pneumonias (IIPs): Identification of potential diagnostic markers and therapeutic targets. BMC Med. Genet..

[55] Miyazaki K (2006). Laminin-5 (laminin-332): Unique biological activity and role in tumor growth and invasion. Cancer Sci..

[56] Plantier L, Crestani B, Wert SE (2011). Ectopic respiratory epithelial cell differentiation in bronchiolised distal airspaces in idiopathic pulmonary fibrosis. Thorax.

[57] Rosmark O, Ibáñez-Fonseca A, Thorsson J (2022). A tunable physiomimetic stretch system evaluated with precision cut lung slices and recellularized human lung scaffolds. Front. Bioeng. Biotechnol..

