# Supplementary material for: Alveolar epithelial cells are competent producers of interstitial extracellular matrix with disease relevant plasticity in a human in vitro 3D model
Source: Sci Rep. 2023 May 31;13:8801. doi: 10.1038/s41598-023-35011-z (PMC10232446; doi:10.1038/s41598-023-35011-z)

Supplementary figure 1 - Flow cytometry gating for evaluation of purity of thawed HT2-280 positive cells

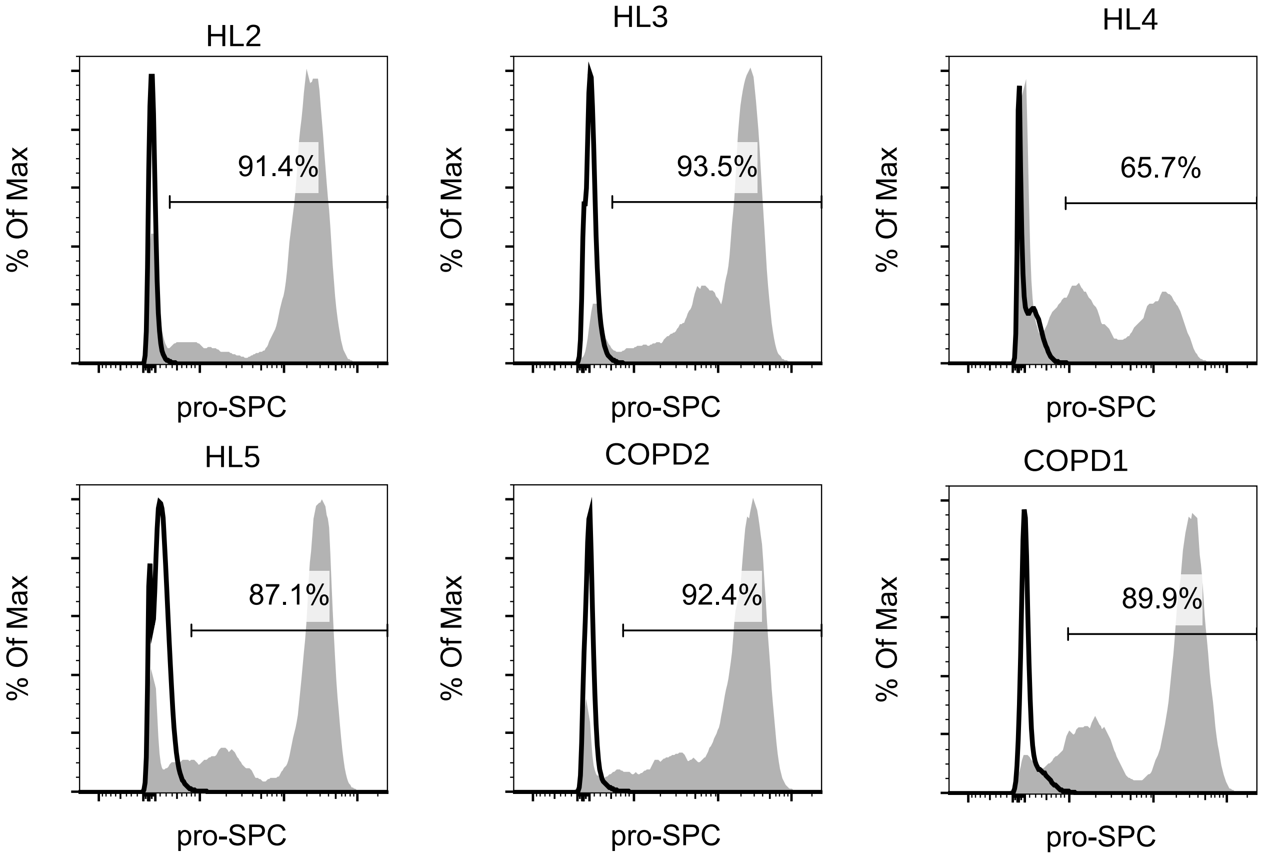

Supplementary figure 2 - Results from linear mixed model of resazurin reduction results

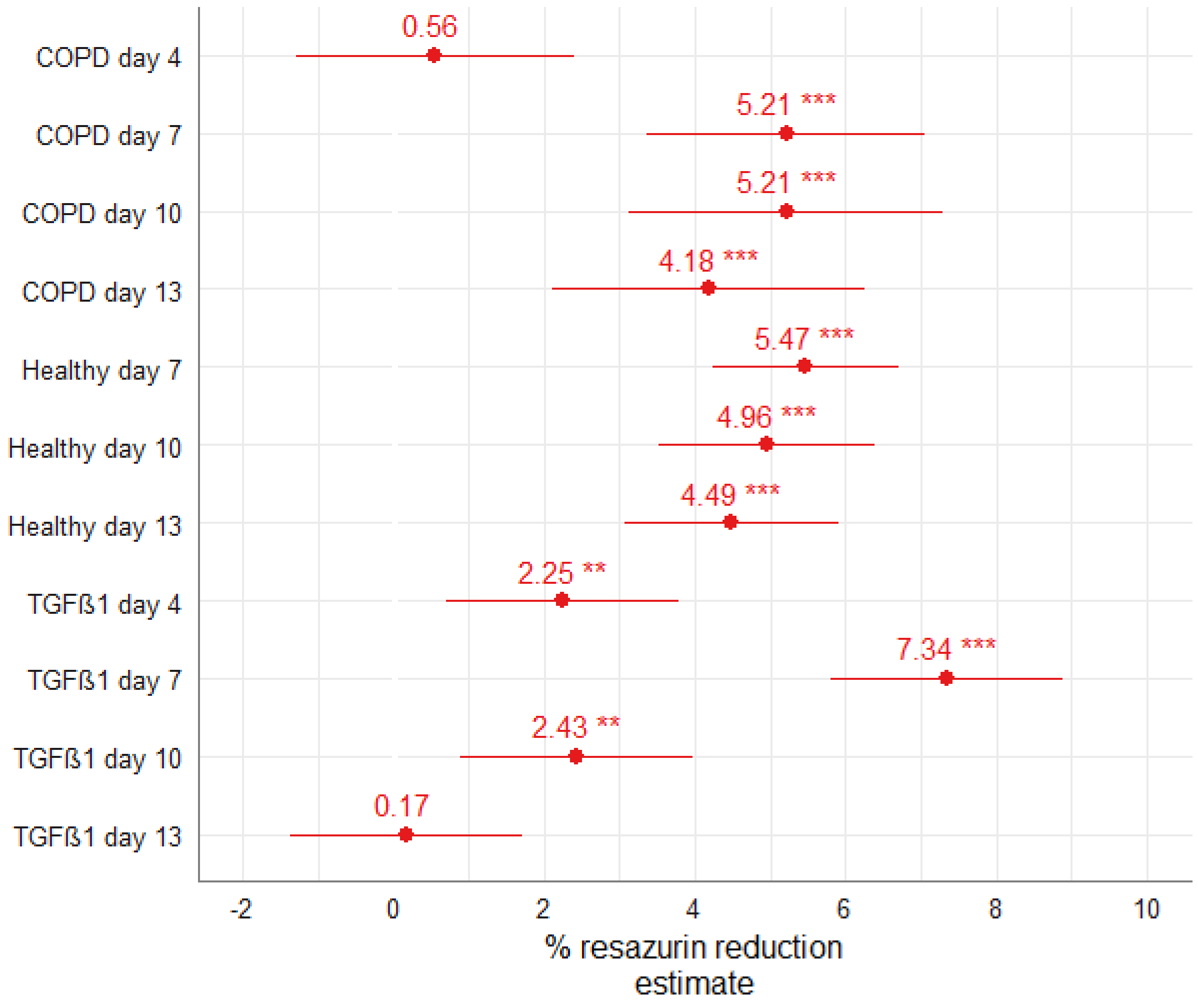

The x-axis show difference from the reference group constituted by Healthy cells day 4 which had a mean reduction percentage of 9.3%. The TGF-ß1 day 4 and day 7 values are from before the TGF-ß1 exposure for started. Error bars display 95% confidence intervals.

Supplementary figure 3 - Haematoxylin and eosin staining of repopulated lung slices after 13 days of culture

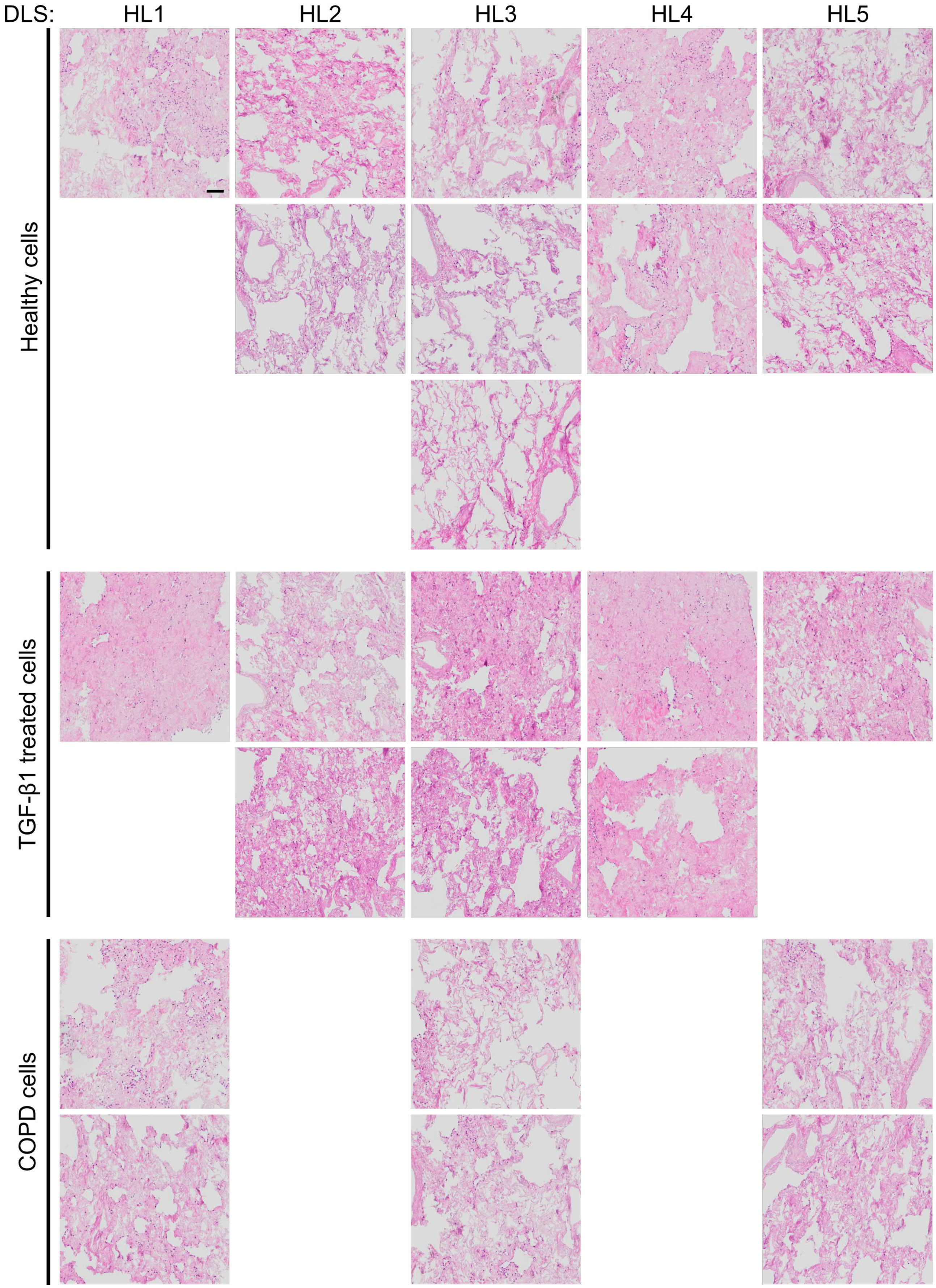

Supplementary figure 4 - Expression of selected EMT markers in cultured AEC cells

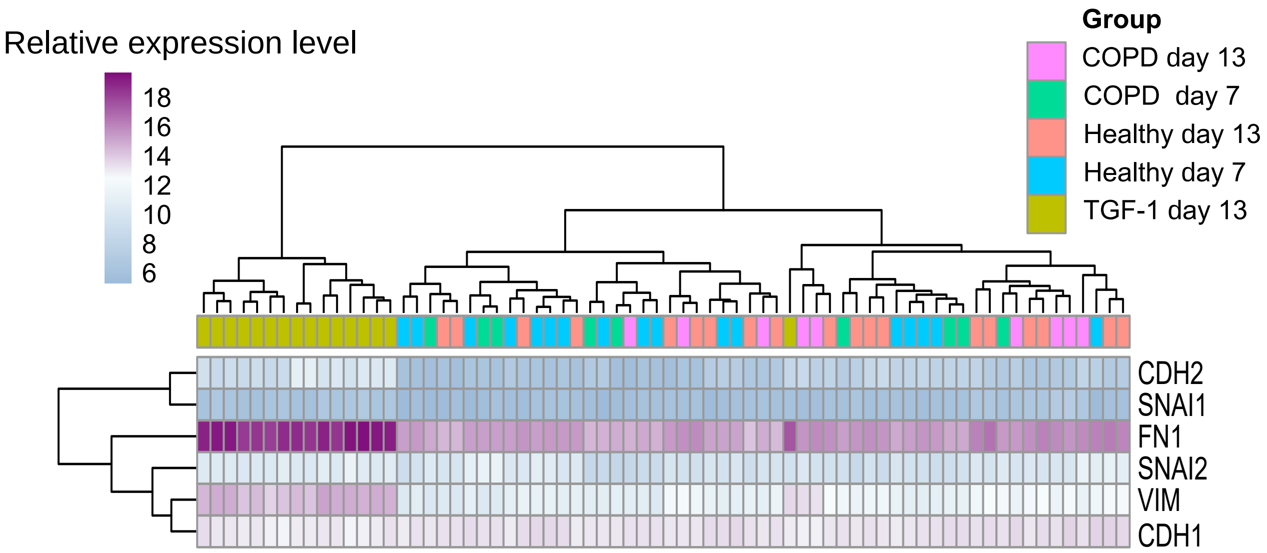

Supplementary figure 5 - Control stainings for surfactant protein B and pro-surfactant protein C related to the stainings in main figure 2-C. Arrowheads point at some of the pro-surfactant protein C positive cells.

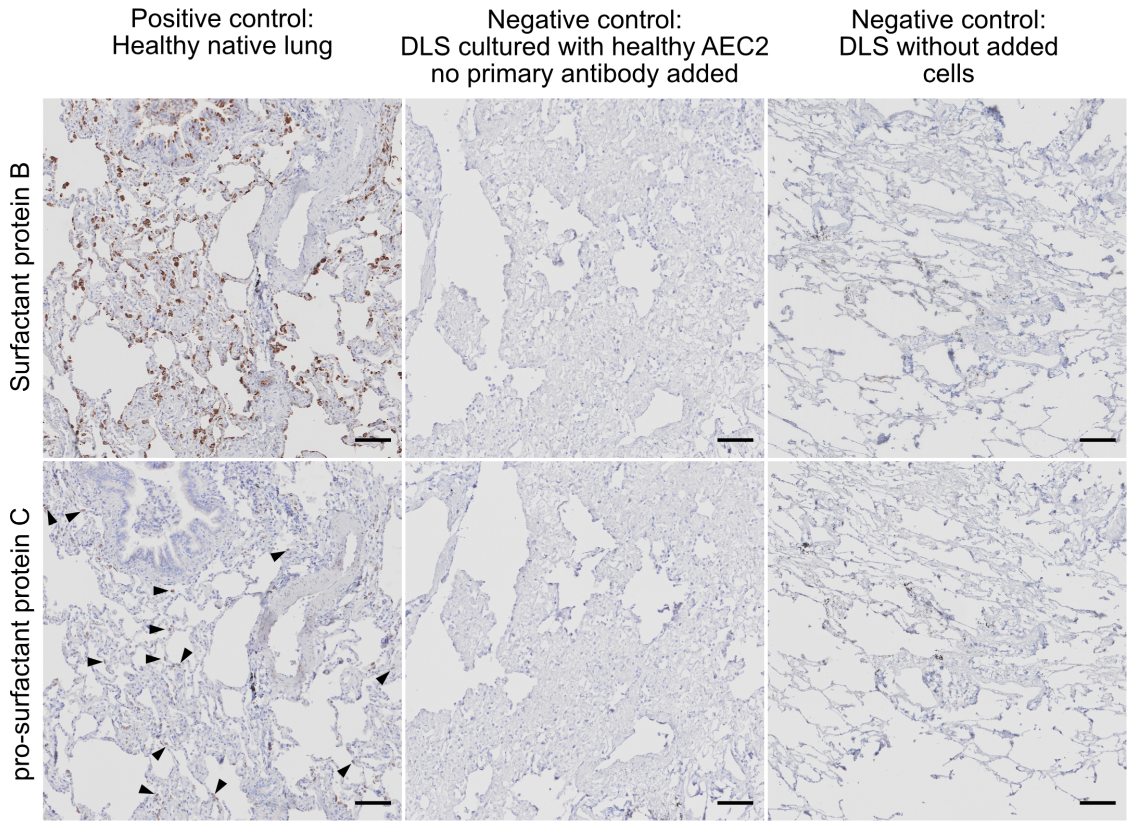

Supplementary figure 6 - IHC stainings for AECI marker caveolin 1 and pan-cytokeratin

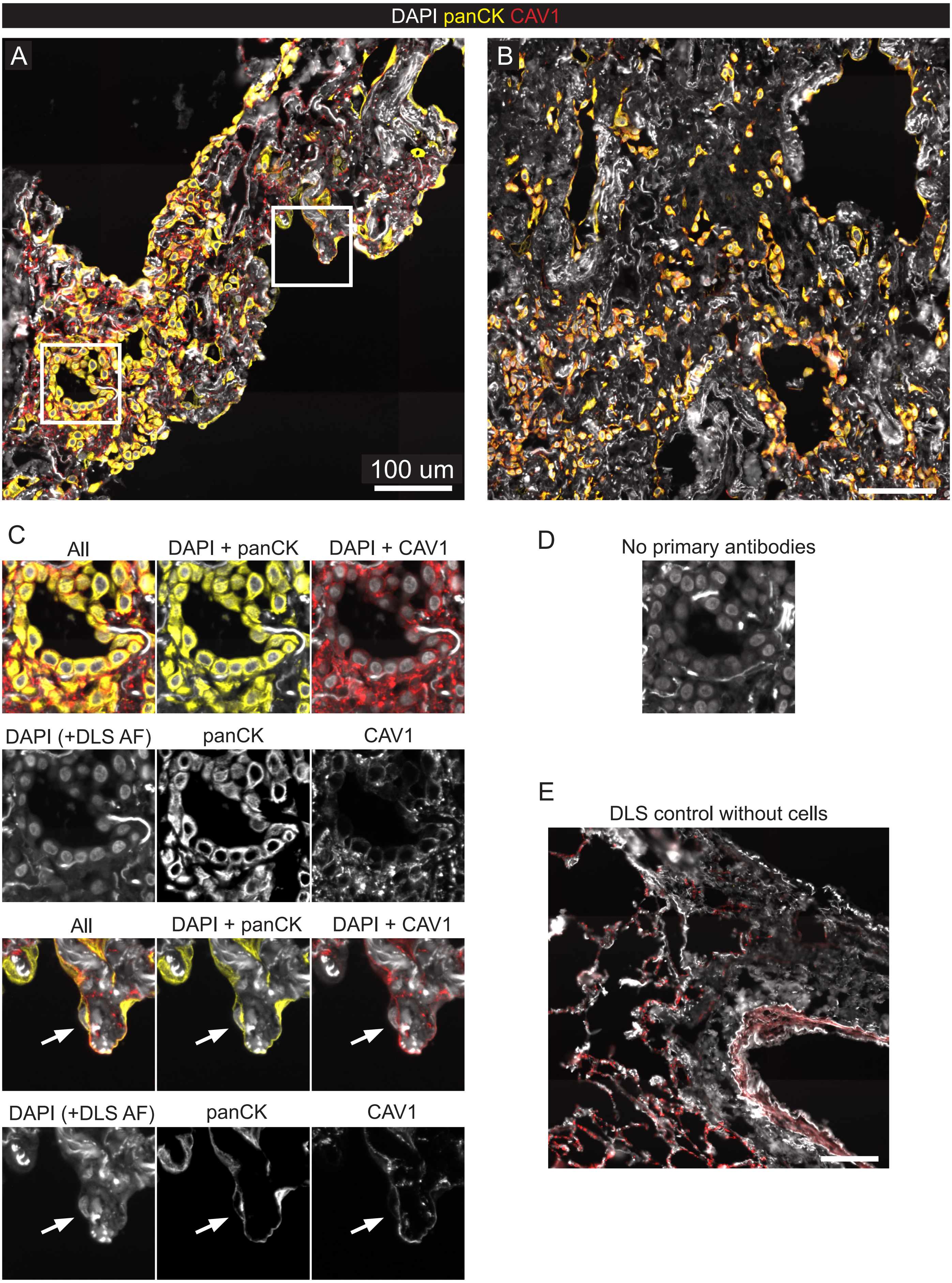

Immunofluorescence co-stainings for pan-cytokeratin (panCK) and the AEC1 marker caveolin-1 (CAV1) in repopulated DLS after 13 days of culture (A and B). Images are from DLS populated with AEC2 from healthy donors (n=3). Magnified quadrant regions (R1 and R2) from A showing examples of CAV1+ cells with cuboidal (R1) and squamous (R2) morphology are displayed with combinations of different channels in pseudocolors and individual channels in grayscale (AF: autofluorescence) (C). Arrows indicate a cell with typical AEC1 morphology in R2. A populated DLS serving as a negative control where primary antibodies were omitted (D). An empty DLS control stained for panCK and CAV1 (D). All scale bars and quadrants are 100 μm.

Supplementary figure 7 - IHC stainings for mesenchymal markers in repopulated lung slices after 13 days of culture. Representative images of stained sections **(A)**, positive staining in red. Results from quantification of stained tissue area for CD90 **(B)** and platelet derived growth factor receptor α (PDGFR-α) **(C)**. n = 2 for native lungs, each datapoint represent one cell donor in **B** & **C**.

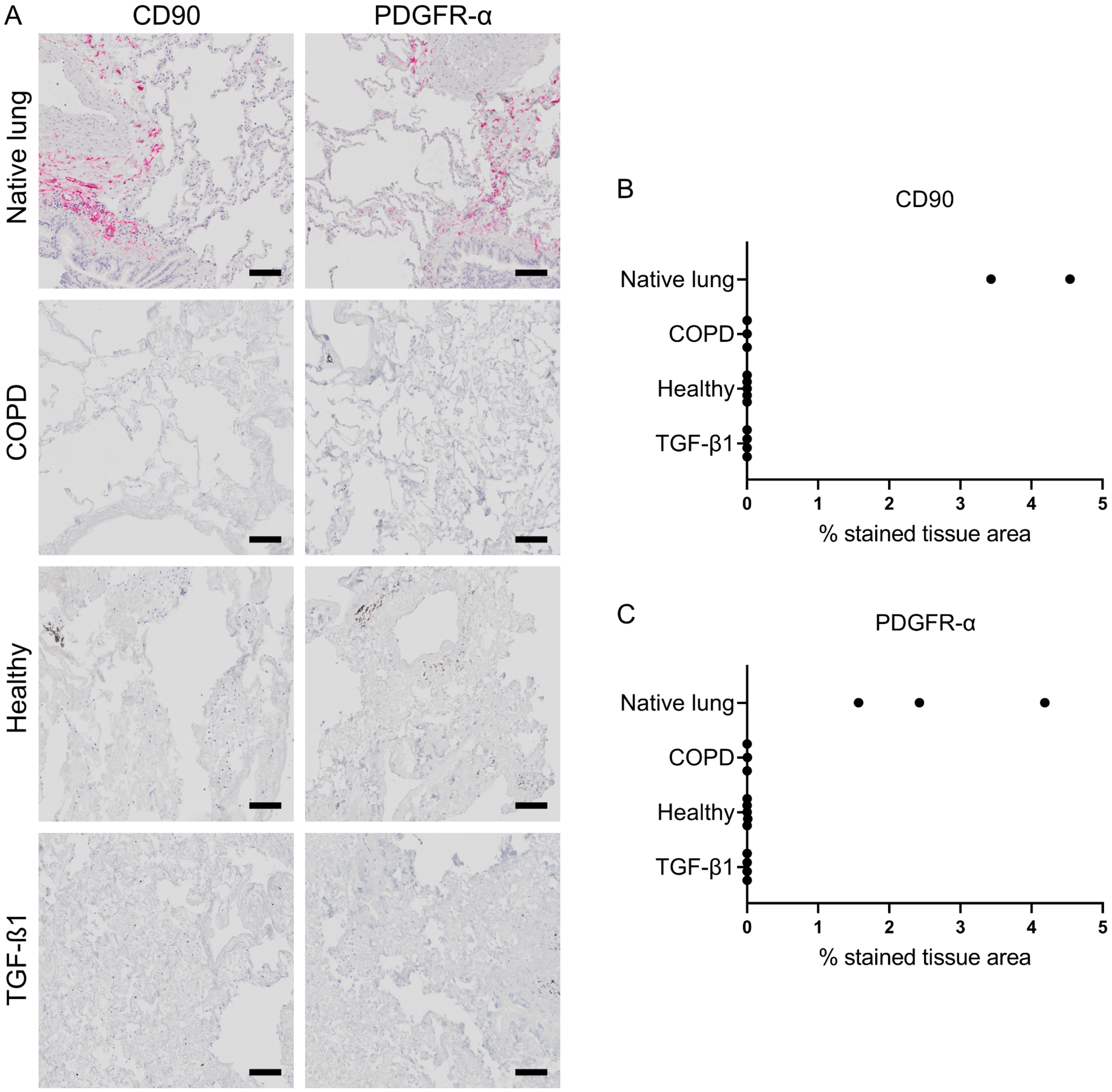

Supplementary figure 8 - Differentially expressed matrisome associated genes in TGF-ß1 treated AEC

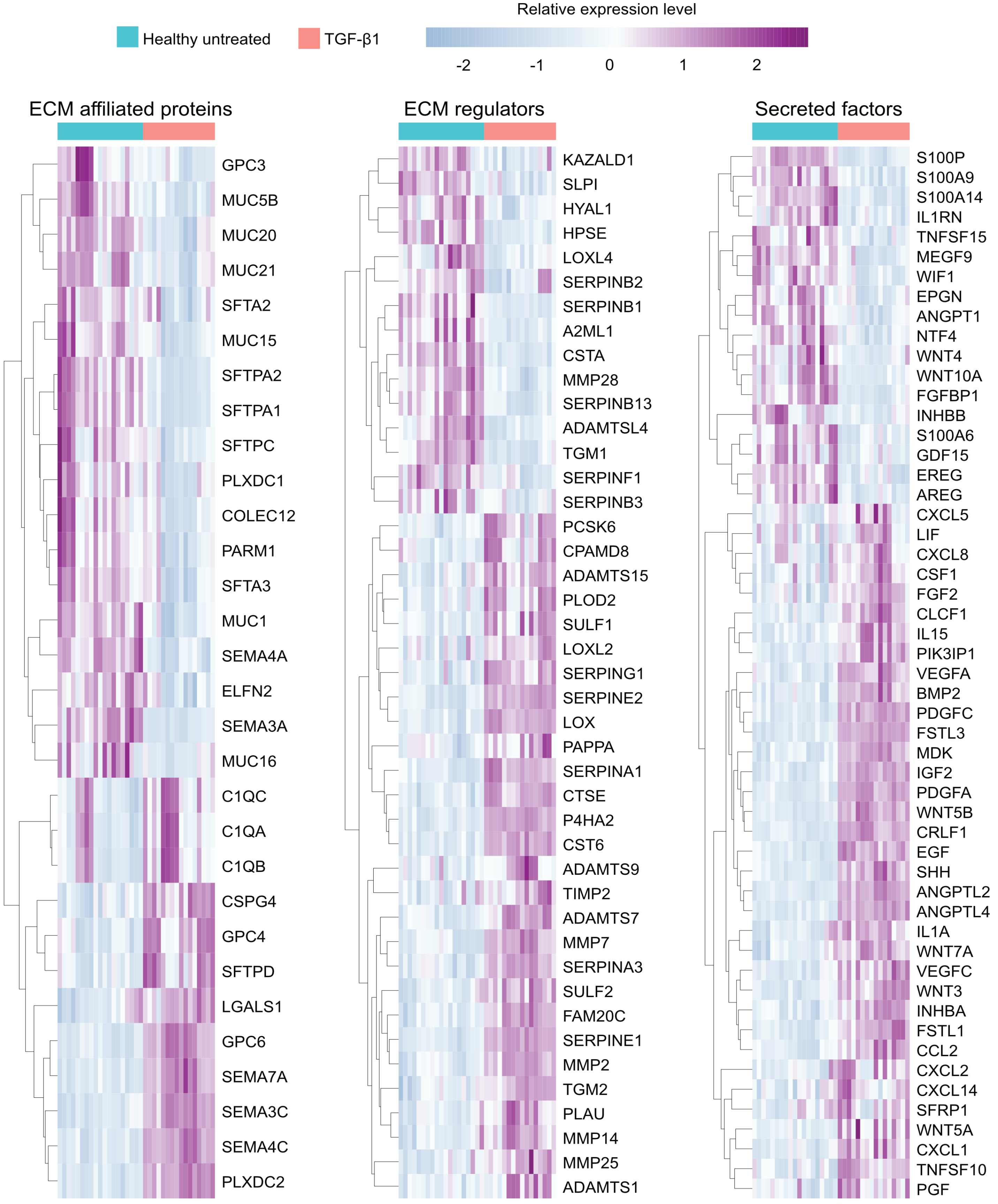

Supplementary figure 9 - Differentially expressed matrisome associated proteins in TGF-ß1 treated AEC

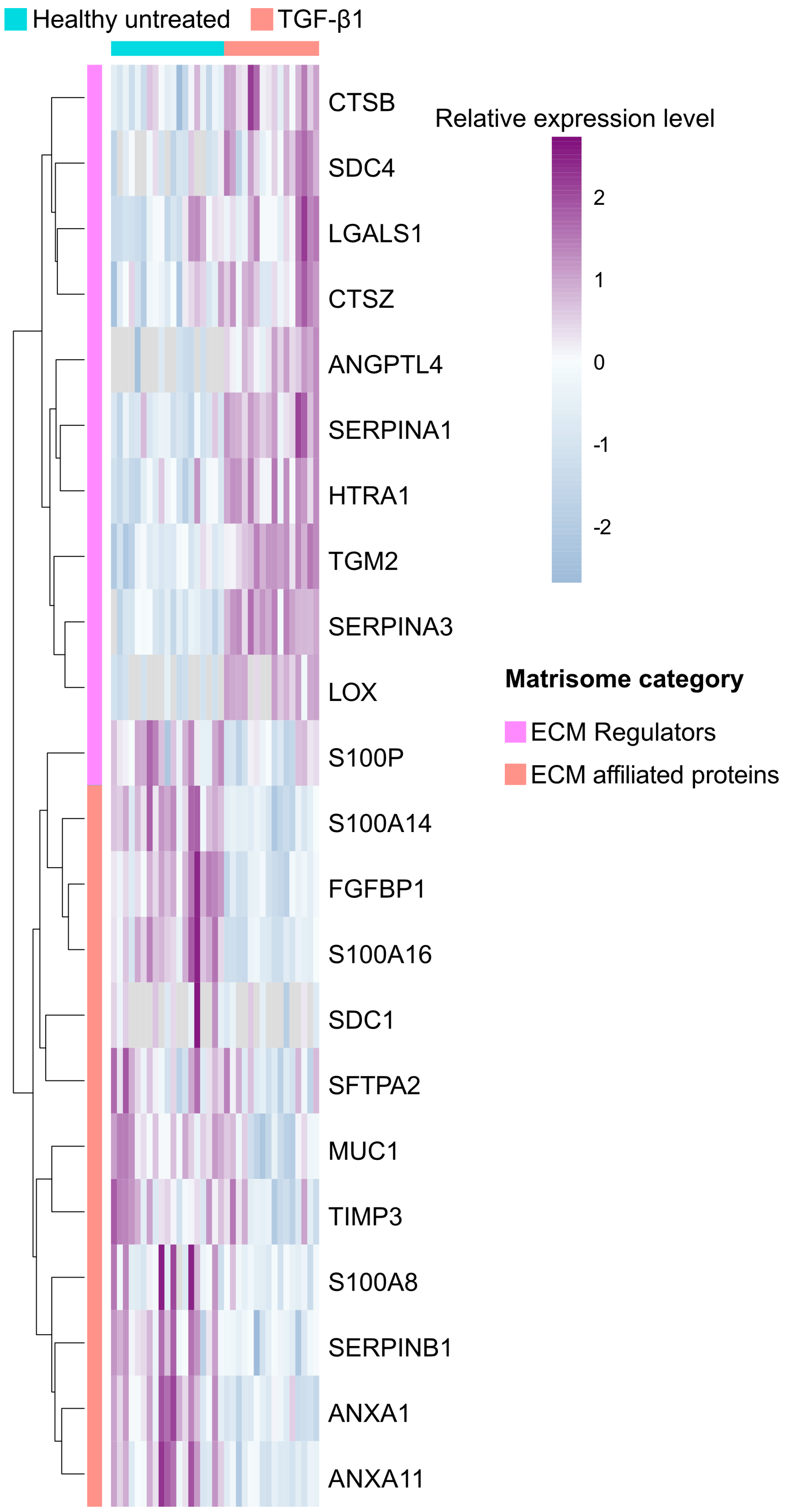

Supplement: Supplementary file 2 — Supplementary Figures. [file 41598_2023_35011_MOESM2_ESM.pdf]
